# Maternal Hypoxia Decreases Capillary Supply and Increases Metabolic Inefficiency Leading to Divergence in Myocardial Oxygen Supply and Demand

**DOI:** 10.1371/journal.pone.0127424

**Published:** 2015-06-01

**Authors:** David Hauton, Abdullah Al-Shammari, Eamonn A. Gaffney, Stuart Egginton

**Affiliations:** 1 Multidisciplinary Cardiovascular Research Centre, University of Leeds, Leeds, United Kingdom; 2 Department of Mathematics, Faculty of Sciences, Kuwait University, Kuwait City, Kuwait; 3 Wolfson Centre for Mathematical Biology, Mathematical Institute, University of Oxford, Oxford, United Kingdom; 4 School of Biomedical Science, University of Leeds, Leeds, United Kingdom; Scuola Superiore Sant'Anna, ITALY

## Abstract

Maternal hypoxia is associated with a decrease in left ventricular capillary density while cardiac performance is preserved, implying a mismatch between metabolism and diffusive exchange. We hypothesised this requires a switch in substrate metabolism to maximise efficiency of ATP production from limited oxygen availability. Rat pups from pregnant females exposed to hypoxia (FIO_2_=0.12) at days 10-20 of pregnancy were grown to adulthood and working hearts perfused *ex vivo*. ^14^C-labelled glucose and ^3^H-palmitate were provided as substrates and metabolism quantified from recovery of ^14^CO_2_ and ^3^H_2_O, respectively. Hearts of male offspring subjected to Maternal Hypoxia showed a 20% decrease in cardiac output (*P*<0.05), despite recording a 2-fold increase in glucose oxidation (*P*<0.01) and 2.5-fold increase (*P*<0.01) in palmitate oxidation. Addition of insulin to Maternal Hypoxic hearts, further increased glucose oxidation (*P*<0.01) and suppressed palmitate oxidation (*P*<0.05), suggesting preservation in insulin signalling in the heart. *In vitro* enzyme activity measurements showed that Maternal Hypoxia increased both total and the active component of cardiac pyruvate dehydrogenase (both *P*<0.01), although pyruvate dehydrogenase sensitivity to insulin was lost (NS), while citrate synthase activity declined by 30% (*P*<0.001) and acetyl-CoA carboxylase activity was unchanged by Maternal Hypoxia, indicating realignment of the metabolic machinery to optimise oxygen utilisation. Capillary density was quantified and oxygen diffusion characteristics examined, with calculated capillary domain area increased by 30% (*P*<0.001). Calculated metabolic efficiency decreased 4-fold (*P*<0.01) for Maternal Hypoxia hearts. Paradoxically, the decline in citrate synthase activity and increased metabolism suggest that the scope of individual mitochondria had declined, rendering the myocardium potentially more sensitive to metabolic stress. However, decreasing citrate synthase may be essential to preserve local PO_2_, minimising regions of hypoxia and hence maximising the area of myocardium able to preserve cardiac output following maternal hypoxia.

## Introduction

Maternal hypoxia (MH) may be one of the most common insults to which the fetus is exposed, yet periods of transient hypoxia are important for tissue development [1], particularly within the heart [2]. Hence, prolonged or severe hypoxia can have profound effects on the fetus, dictated by the period during development when the hypoxic insult occurs [3]. We have previously shown in rats that MH decreased capillary density (CD) in the hearts of adult offspring when the period of hypoxia corresponded to gestational days 10–20 (E10-20), and we demonstrated an increase in left ventricular developed pressure [4]. These data suggest that a mismatch between metabolism and oxygen consumption may result, i.e. increased cardiac work coupled with a decrease in CD (the latter indicating a reduction in the potential for diffusive exchange). However, such investigations were performed in glucose-perfused Langendorff heart preparations. Whilst exceptionally useful for investigating cardiac mechanical performance, this approach is not representative of the physiological performance of the heart *in vivo* as it measures isometric contraction, i.e. tension developed in the left ventricular wall when contracting against a fluid-filled (non-compressible) balloon. Isometric contraction represents only a small part of the cardiac cycle and is less suitable for quantifying substrate metabolism. Therefore, to investigate more fully the mechanical and metabolic changes arising from MH we utilised the perfused ‘working’ heart, which provides a more physiological preparation examining the entire cardiac cycle.

Only modest effects of MH on recovery from ischaemia-reperfusion (IR) injury have been previously noted. Hearts from both male and female adult rats exposed to MH on E15-21 showed diastolic dysfunction *in vivo*, with male rats also demonstrating left ventricular hypertrophy at 4 months of age [5]. For the *ex vivo* perfused heart mechanical performance was normal following MH, yet recovery from mild ischaemia was impaired with hearts demonstrating an increased LV end-diastolic pressure, decreased developed pressure and decreased coronary flow [6]. In addition, the resulting infarct size was also increased as a consequence of MH [6]. Similar decreases in functional recovery were noted by others following IR of MH hearts from 4- and 7-month old rats [7].

The few studies that have investigated the metabolic characteristics of MH hearts suggest that metabolic changes are also modest. During the pre-ischaemic period of ischaemia-reperfusion experiments metabolism was unaffected by MH compared with age-matched controls, while aerobic cardiac efficiency—measured as production of acetyl-CoA per unit cardiac work—was significantly decreased in both male and female offspring [8]. However, under conditions of metabolic stress (IR injury), changes in both metabolism and function once more become manifest, and were more marked in male offspring of MH rats, showing increased rates of glycolysis coupled with increased proton production [8]. However, the period of MH within all these experiments was relatively short (E15-21), while our experiments exploited a more severe form of MH covering E10-20. Although none of the previous studies have presented evidence for altered capillary density (CD) we have demonstrated that for this, more severe stress, MH likely reduces microvascular diffusive exchange.

Given the pivotal role that diffusive exchange plays for both oxygen and substrates in controlling metabolism [9], we postulate that the decreased capillary surface area for oxygen diffusion will favour the metabolism of glucose over fatty acids as metabolic fuel for the myocardium. As the synthesis of ATP from available oxygen sources is more efficient (more ATP produced) under such conditions, we predict that facilitating glucose metabolism through addition of insulin (to increase glucose uptake) will further support cardiac work. We quantified the effect of decreased CD associated with MH on metabolism in the perfused, working rat heart utilising both glucose and palmitic acid, at physiologically-appropriate concentrations, to investigate the substrate preference and mechanical performance following exposure to the physiological challenge of maternal hypoxia.

## Materials & Methods

### Materials

^3^H-[9,10]-palmitic acid and [U-^14^C] glucose were purchased from Amersham Biosciences (Chalfont, UK); palmitic acid, glucose, fatty acid-free bovine albumin, and all buffer reagents were obtained from Sigma (Poole, UK). All solvents were ANALAR grade and purchased from Fisher Scientific (Loughborough, UK).

### Methods

#### Animal maintenance

All experiments were carried out in accordance with the UK Home Office, Animal (Scientific Procedures) Act 1986 and the experiments were approved by the University of Birmingham, College of Medical and Dental Sciences Ethical Review Committee. Animals were housed at 22°C 12hr light/12hr dark with *ad libitum* access to food and water (RM3 rat chow, Lillico Biotechnology, UK) and water throughout the experiment. After confirmation of mating, female Wistar rats (200g; Charles River, UK) were housed singly in a ventilated chamber breathing room air (FIO_2_ = 0.2) for the first 10 days of pregnancy. Animals were then exposed to a normobaric hypoxic atmosphere (12% oxygen, balance nitrogen: FIO_2_ = 0.12) for days 10–20 of pregnancy. During this time the enclosed atmosphere was circulated through silica gel and soda lime to trap water vapour and carbon dioxide, respectively. At 20 days post-mating the animals were transferred to room air and the pregnancy progressed as normal. At birth, pups remained with the mother and were weaned normally at 4 weeks of age, after which the pups were divided by gender and housed with littermates in groups (n = 5). These litters were then maintained for a further 10 weeks in normal room air with *ad libitum* access to both food and water.

#### Experimental groups

All experimental groups contained 6 rats matched for age (14 weeks from birth). The MH rats (n = 6) were obtained from six separate litters of pups and selected at random (i.e. one per litter). Animals were fasted overnight prior to experimentation to remove confounding effects of diet and insulin, and experiments commenced before 10am.

#### Perfused working heart

Working hearts were perfused as previously described [10]. Briefly, anaesthesia was induced with isoflurane (4% in oxygen) and following thoracotomy hearts were excised and the aorta cannulated (16G cannula), then perfused initially in retrograde fashion [11]. The left atrial opening was then cannulated and the cannula secured with a silk suture. Hearts were maintained at 37°C and perfused with a Krebs-Henseleit crystalloid medium (KH) supplemented with glucose (5mM) and CaCl_2_ (1.3mM) gassed with O_2_/CO_2_ (95:5). Atrial filling pressure was fixed at 10cm H_2_O with afterload fixed at 100cm H_2_O. Control hearts were perfused with glucose (5mM supplemented with U-^14^C-labelled glucose 0.185MBq/perfusion) and palmitic acid (0.4mM pre-bound to bovine albumin + ^3^H palmitic acid 5.55MBq/perfusion). All hearts were unpaced. Metabolism was estimated from timed collection of perfusate and effluent gases (see below) for 60min to quantify utilisation of glucose and palmitate. For selected experiments hearts were supplemented with bovine insulin. All insulin solutions were made fresh for each experiment from a stock dissolved in acidified saline prior to immediate dilution to give a final insulin concentration of 100mU/litre on addition to the perfusate. At termination of the experiment hearts were frozen to estimate heart metabolite concentrations. For estimates of capillary density another group of fresh hearts were collected, in order to preserve the insulin-treated tissues for metabolic investigations.

#### Quantitation of plasma tritiated water

For selected experiments, metabolism of palmitate was estimated from quantitation of tritiated water production, as previously described [10]. Briefly, aliquots of perfusate (1.0ml) were extracted with chloroform:methanol (2:1) and metabolism estimated in the aqueous fraction by scintillation counting. Metabolism was calculated with reference to specific activity at the start of the experiment.

#### Glucose metabolism

For selected hearts perfusate glucose was supplemented with U-^14^C-labelled glucose (as above). Effluent gases were collected from a gas-tight perfusion apparatus into ethanolamine/ethylene glycol (2:1) solution [12] and samples of perfusate were recovered to estimate the liberation of ^14^C-labelled CO_2_ as CO_2_ or bicarbonate, as previous detailed [10].

#### Lactate flux

Aliquots of perfusate were treated with perchloric acid (PCA; 0.6N) to deproteinise the sample and stored for analysis later. For neutralised PCA-treated perfusate samples lactate was estimated enzymatically, following the conversion of lactate to pyruvate and quantifying NADH absorbance at 340nm [13]. Rates of lactate accumulation were estimated from timed collections of perfusate, and linear regression analysis of lactate synthesis used to estimate the rate of lactate production.

#### Tissue glycogen concentration

Total cardiac glycogen and incorporation of ^14^C-labelled glucose into tissue glycogen were estimated as previously outlined [14]. Briefly, cardiac tissue (∼50 mg) was digested in alkali (200μl, 30% w/v KOH, 70°C, 60minutes). Glycogen was precipitated from the resulting digest after addition of 5 vol ice-cold absolute ethanol. After centrifugation, the pellet was resuspended in water, and the precipitation was repeated. Glycogen pellets were air-dried and re-dissolved in acetate buffer (50 mmol/L, pH = 4.5). ^14^C-labelled glucose incorporation was estimated after scintillation counting of an aliquot of the re-dissolved glycogen. The remainder of the glycogen was treated with amyloglucosidase (100 units per reaction; final volume, 0.5 ml) and digested overnight. Liberated glucose was estimated spectrophotometrically by the glucose oxidase method.

#### Total glucose uptake

To calculate the total uptake of glucose the assumption was made that glucose underwent oxidation, metabolism to lactate, or storage as glycogen. Therefore, total glucose uptake was calculated as: (glucose oxidation rate) + (net glycogen synthesis rate) + (lactate synthesis rate/2).

#### Estimates of acetyl-CoA synthesis

In order to estimate ‘total metabolism’, rates of acetyl-CoA synthesis were calculated from the rates of metabolism for glucose and palmitate measured from the perfusate. Briefly, estimates were based on the assumption that complete metabolism of 1 mole of glucose yields 2 moles acetyl-CoA, and complete metabolism of 1 mole palmitate yields 8 moles acetyl-CoA [5].

#### Cardiac efficiency calculations

Cardiac efficiency was calculated from acetyl-CoA synthesis rate (nmoles/min) divided by cardiac output (ml/min) to estimate acetyl-CoA consumption per unit volume of cardiac output. These data were then corrected for cardiac mass.

#### Citrate synthase activity

Citrate synthase activity was measured spectrophotometrically by the method of Morgunov and Srere [15]. Activity was estimated from the reduction of DTNB in the presence of oxaloacetate, followed at 412nm. Rates of reaction were expressed relative to homogenate protein content. Protein concentrations were estimated using BCA protein assay (Sigma, Poole, UK).

#### Acetyl-CoA carboxylase (ACC) activity

Cardiac ACC activity was estimated by the *in vitro* bicarbonate fixation method of Saddik *et al*. [16]. Rates of reaction were estimated from fixing of ^14^C-labelled bicarbonate into malonyl-CoA. Radioactivity was estimated in the aqueous fraction by liquid scintillation counting. Homogenate protein concentrations were estimated using BCA protein assay (Sigma, Poole, UK).

#### Pyruvate dehydrogenase activity

Pyruvate dehydrogenase activity was measured using the method of Seymour and Chatham [17]. Briefly, two components of pyruvate dehydrogenase (PDH) activity were isolated, total and active. Activity was estimated from the reduction of NAD, and rates of reaction were followed spectrophotometrically at 340nm.

#### Capillary density

Tissues were mounted onto cork disks (22mm: R.A. Lamb, Eastbourne, East Sussex, UK) in Tissue-Tek OCT compound (Sakura, Torrance, CA) before freezing in liquid nitrogen-cooled isopentane. Cryostat sections (10μm) were cut and fixed onto glass slides. Capillaries were visualised using alkaline phosphatase activity (Sigma) using a Zeiss Axioskop microscope [18]. Vessel density was quantified from digital images (magnification x200) in regions of known area for four non-consecutive sections using Image J image analysis software (NIH). A minimum of six fields were counted per section, and three sections for each heart were quantified. Sections were counted at random and the viewer was blinded to the origins of the tissue. Data were expressed as capillary profiles/mm^2^ cross-sectional area.

#### Cardiac fibrosis

Selected heart tissue sections were stained with Masson’s Trichrome and picrosirius red, as previously described [19,20], to quantify collagen infiltration and fibrosis using stereological point-counting techniques: area percent = (Pi/Pt) x100, where Pi is the number of points from a square grid lying over collagen and Pt is the total number of points lying over the tissue.

#### Oxygen diffusion calculations

From histological images of perfused hearts, the shortest distance between adjacent capillaries was calculated, to determine ‘nearest neighbours’ [21], and by calculation, the variance in capillary supply between the normal state and following MH were determined. Voronoi tessellation of digitised images was used to produce polygons centred on each individual vessel, the boundaries between two polygons representing the lowest oxygen concentration between adjacent capillaries [22]. By calculation, capillary density, domain area, and nearest neighbour distance (NN) as standard deviation/mean x100 were estimated. PO_2_ heat maps were generated as detailed Al-Shammari et al. [23] modelling oxygen consumption using Michaelis-Menton kinetics [9]. Measured citrate synthase activities were used to estimate tissue oxygen consumption in control and MH hearts. Subsequently, calculations were repeated using measured domain areas with maximum predicted oxygen consumption for mitochondria to estimate the size of regions of hypoxia under conditions of maximum oxygen extraction.

#### Proteomics analysis: Gel electrophoresis

Briefly, cardiac tissue (50 mg) was powdered in liquid nitrogen and extracted with radioimmunoprecipitation assay (RIPA) buffer containing protease and phosphatase inhibitors, followed by centrifugation (10,000 rpm for 10mins) and recovery of the supernatant. Samples were diluted with 5x sample buffer (containing mercaptoethanol as reducing agent) to give a final protein concentration of 2mg/ml. Samples (40μg) were loaded onto a reducing SDS-PAGE gel and eluted at constant current. Bands stained with Coomassie Blue were identified visually using a lightbox and regions of interest were excised from the gel with a sterile scalpel.

#### Sample trypsinisation

Coomassie bands were excised, divided (~2 mm^3^ cubes) and de-stained with acetonitrile followed by ammonium bicarbonate (100mM). De-stained gel pieces were dried (vacuum centrifugation; 5 min) and rehydrated in DTT (10mM), ammonium bicarbonate (100mM). Gel fragments were repeatedly washed with ammonium bicarbonate (100mM). Hydrolysis of peptides (20μg trypsin gold; Promega, WI, USA) occurred overnight (~16 h) at 37°C. Peptides were extracted with the initial solution of 2% (w/v) acetonitrile, 0.1% (w/v) formic acid in water was added and shaken for 30 minutes. A second peptide extraction was performed using 40% (w/v) acetonitrile, 0.1% (w/v) formic acid in water, shaken for 30 mins at room temperature. The supernatants were pooled and dried in an evaporator, then re-suspended in 0.1% (w/v) formic acid/water in preparation for the mass spectrometry analysis.

#### Mass spectrometry analysis

UltiMate 3000 HPLC series (Dionex, Sunnyvale, CA USA) was used for peptide concentration and separation. Samples were separated in Nano Series Standard Columns (75 μm i.d. x 15 cm) packed with C18 PepMap100 (3 μm, 100Å). The mass spectrometer alternated between a full FT-MS scan (m/z 380–1600) and subsequent collision-induced dissociation (CID) MS/MS scans of the 7 most abundant ions. The MS and MS/MS scans were searched against NCBInr database using Mascot algorithm (Matrix Sciences) and software Proteome Discoverer 1.3 (ThermoFisher Scientific, Germany) to identify candidate peptides.

#### Statistical analysis

All data are presented as mean ± standard deviation. Curve fitting analysis and estimation of mathematical functions was undertaken with appropriate computer software (CurveExpert 1.4–2009). For the proteomics data raw peak intensities were averaged and this value used to calculate standard deviation in the control group. Mean peak intensity for control hearts was assigned ‘unity’ and fold increases for MH hearts calculated. Statistical significance between individual groups was calculated using ANOVA analysis where *P*<0.05 was taken to indicate statistical significance.

## Results

### Animal phenotype

**Fig 1:**
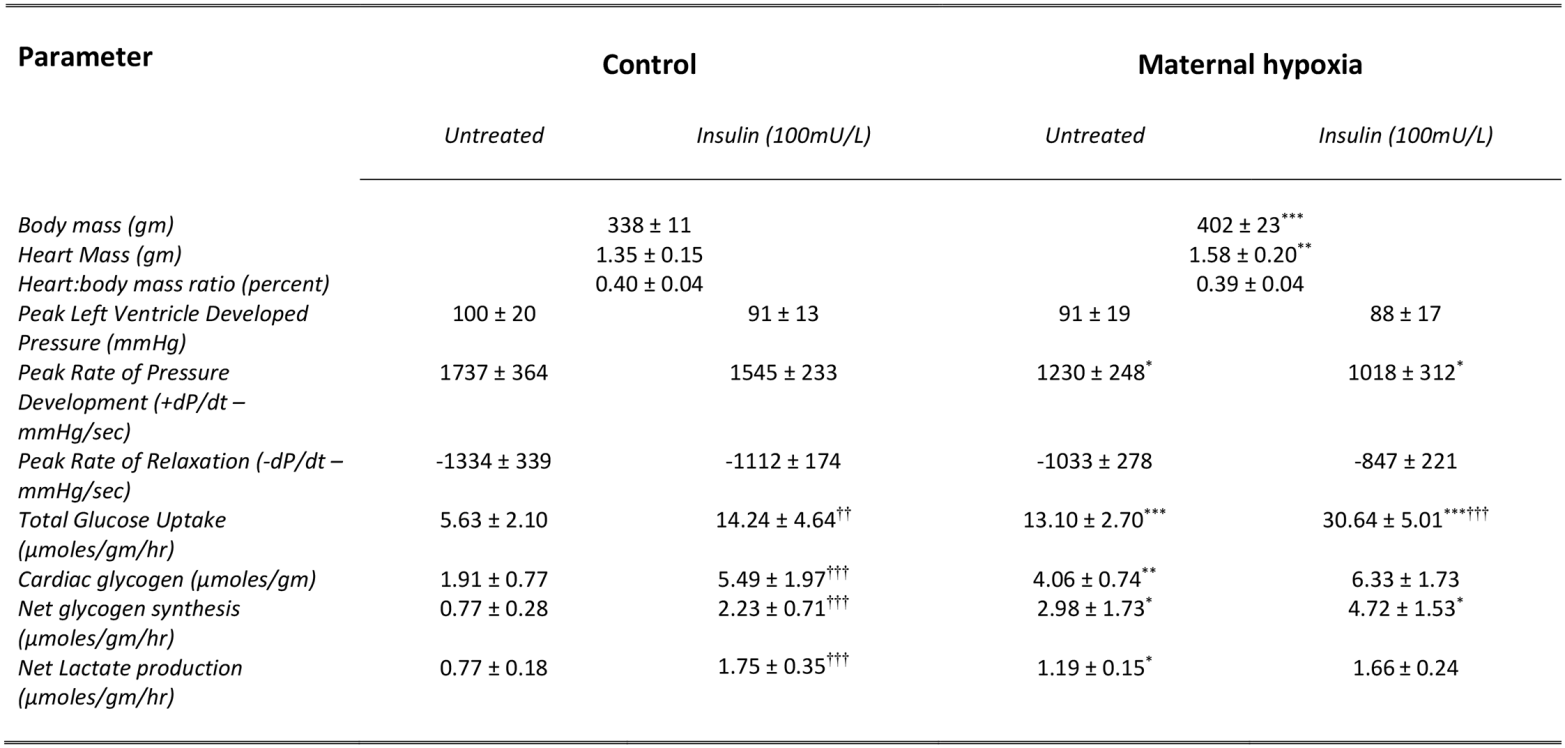
The effect of maternal hypoxia on adult offspring at post-mortem and cardiac parameters for perfused hearts. Control and maternal hypoxia (MH) rats (age = 14weeks) were anaesthetised and hearts excised and perfused as detailed in the methods section. Data represents Mean ± SD (n = 6). Statistical significance represented as: effects of maternal hypoxia * P<0.05, ** P<0.01, *** P<0.001: effects of insulin †† P<0.01, ††† P<0.001.

Following weaning, maternal hypoxia (MH) rats gained weight at an accelerated rate over the 14 weeks following birth leading to a 20% increase in body mass compared with controls (*P*<0.01; Fig 1), accompanied by a corresponding increase in cardiac mass (*P*<0.01; Fig 1). Fibrosis in the heart, estimated from area-percent measurements revealed ~5% of control myocardium stained as picrosirius red-positive fibres, with MH leading to a modest (20%) increase in positive-staining fibres (*P*<0.05). Estimates of capillary density (CD) using alkaline phosphatase-positive staining showed a 20% decrease in capillary supply (*P*<0.01) for left ventricular free wall following MH. Gel electrophoresis of whole heart homogenates indicated no change in the proportions of myosin heavy chains α and β present in the MH-heart (NS).

### Mechanical performance

**Fig 2:**
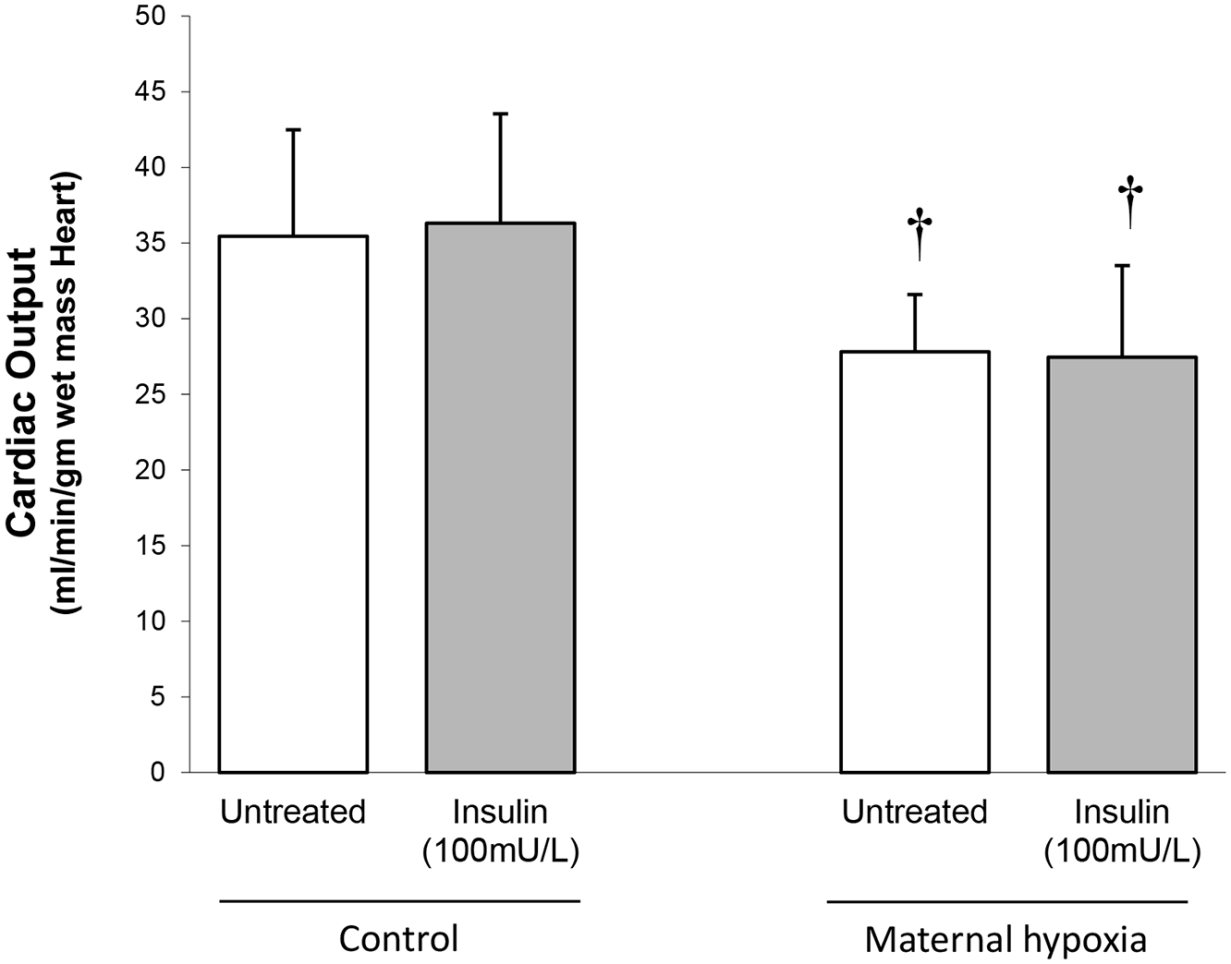
Cardiac output measured in perfused working heart from control and adult offspring of rats exposed to maternal hypoxia (MH). Unpaced hearts were perfused at constant preload (10cmH_2_O) and afterload (100cm H_2_O), cardiac output was estimated from aortic flow and coronary flow, and flow rates were estimated from timed recovery of known perfusate volumes. Data represents mean ± SD (n = 6). Statistical significance is indicated: effects of maternal hypoxia † *P*<0.05.

Perfused hearts from MH rats showed a 20% decrease in cardiac output when compared with controls (*P*<0.05; Fig 2). There was no change in peak developed pressure produced by perfused hearts from MH rats (NS; Fig 1), however peak rate of pressure development (+dP/dt) was significantly decreased by 30% compared with controls (*P*<0.05; Fig 1). Rates of left ventricular relaxation (estimated from–dP/dt) were unchanged by MH (NS; Fig 1).

### Cardiac metabolism

**Fig 3:**
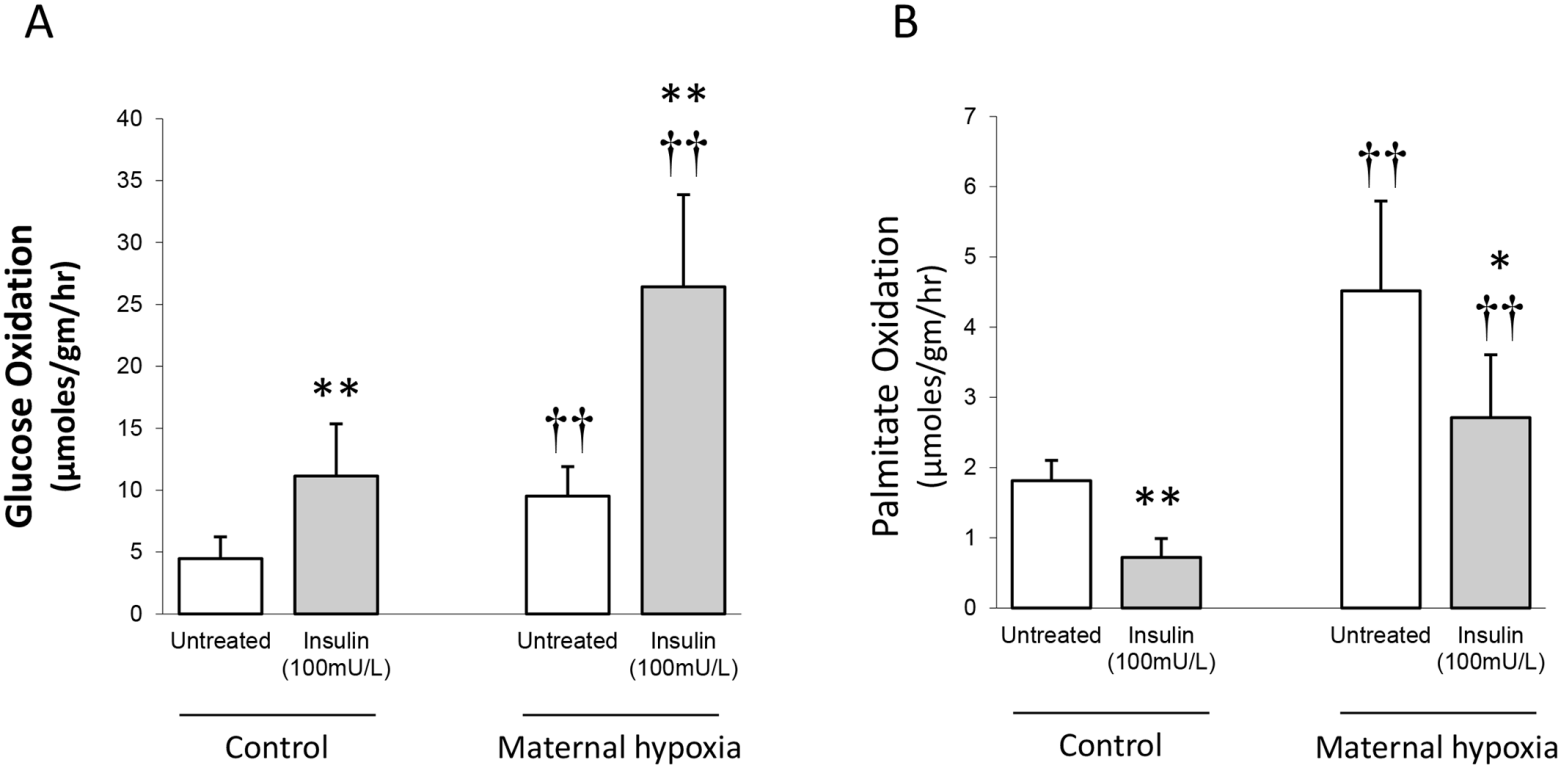
Substrate metabolism in the isolated perfused control and MH heart. Hearts were perfused with ^14^C-labelled glucose and ^3^H-palmitic acid and metabolism estimated from recovered ^14^CO_2_ and ^3^H_2_O, respectively. (A) Glucose oxidation was estimated in the absence and presence of insulin (100mU/L). (B) Palmitate oxidation was estimated in the absence and presence of insulin (100mU/L). Data represents mean ± SD (n = 6). Statistical significance is indicated: effects of maternal hypoxia †† *P*<0.01; effects of insulin * *P*<0.05, ** *P*<0.01.

Oxidation of glucose (Fig 3A) and palmitate (Fig 3B) by perfused hearts were increased 2-fold by MH, compared to control rats (both *P*<0.01). Addition of insulin to hearts from control, fasted rats doubled the rate of glucose oxidation (*P*<0.01; Fig 3A) and this was accompanied by a two-thirds decrease in palmitate oxidation (*P*<0.01; Fig 3B). Addition of insulin to MH-hearts increased rates of glucose oxidation 2.5-fold (*P*<0.01; Fig 3A) and approximately halved the rate of palmitate oxidation compared with fasted MH-hearts (*P*<0.01; Fig 3B).

MH doubled the cardiac content of unlabelled glycogen when compared with controls (*P*<0.01; Fig 1). Addition of insulin to fasted control hearts increased unlabelled glycogen 2.5-fold (*P*<0.001; Fig 1), yet for MH hearts had no effect on glycogen content (NS; Fig 1). Net synthesis of glycogen, estimated as the incorporation of ^14^C-labelled glucose into glycogen, was 4-fold higher in MH hearts compared with fasted control hearts (*P*<0.001; Fig 1). Insulin treatment of fasted control hearts led to a 3-fold increase in labelled glucose incorporation into glycogen (*P*<0.001; Fig 1), but for MH hearts insulin had no effect (NS; Fig 1). Fasted-MH rat hearts showed a significant increase in net lactate production, measured as accumulation of lactate in the perfusate, when compared to fasted control rats (*P*<0.05; Fig 1). Addition of insulin to fasted control hearts led to a 2-fold increase in the release of lactate (*P*<0.05; Fig 1), however similarly-treated MH hearts showed no change in net lactate production rates (NS; Fig 1). For control hearts, insulin increased total glucose uptake 2.5-fold (P<0.01; Fig 1). MH rats showed double the glucose uptake compared to control rats (P<0.001; Fig 1) and insulin treatment of MH-hearts led to a 2.5-fold increase in glucose uptake (P<0.001; Fig 1).

### Pyruvate dehydrogenase activity

**Fig 4:**
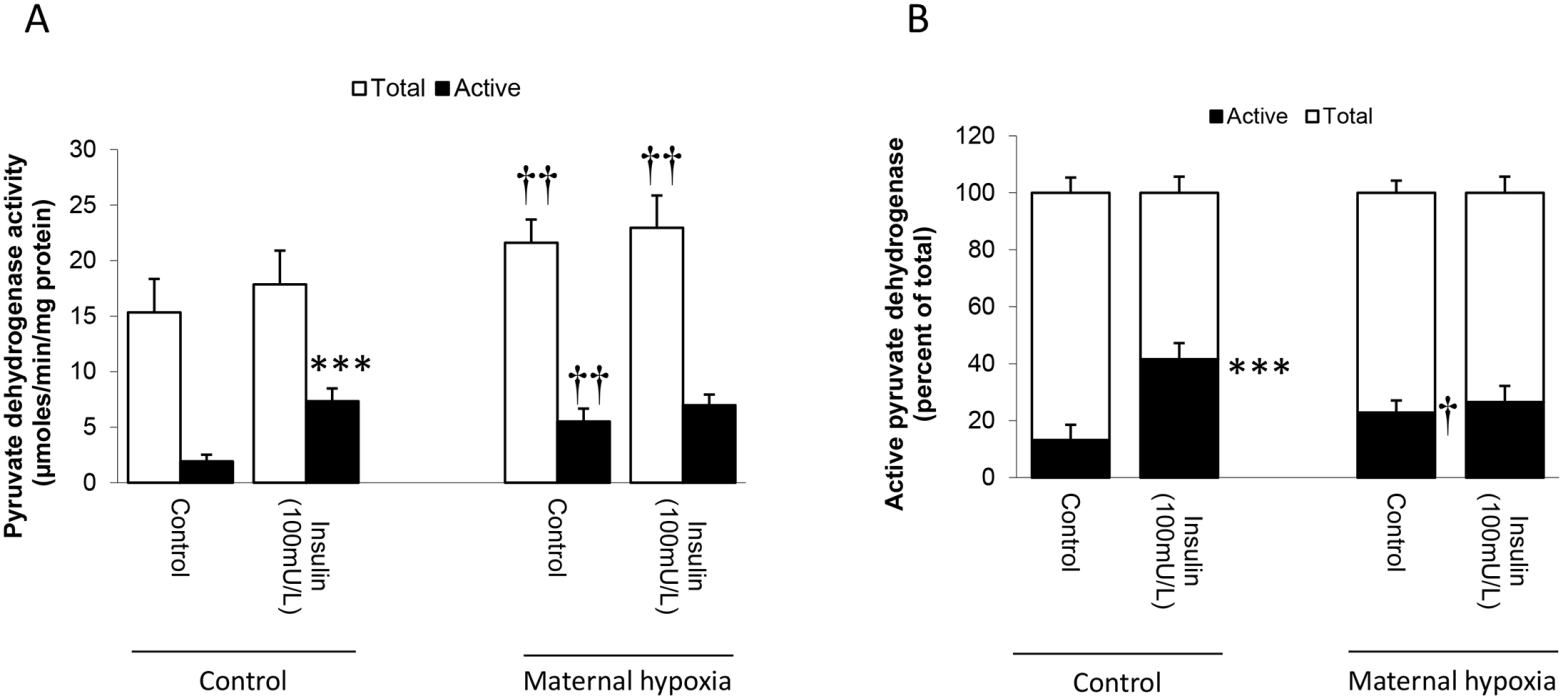
Maternal hypoxia and effects on cardiac pyruvate dehydrogenase activities. Effects of maternal hypoxia on absolute rates of pyruvate dehydrogenase activity (A) and the proportion of pyruvate dehydrogenase that formed the active component (B). Pyruvate dehydrogenase enzyme was isolated from heart homogenates from control and MH hearts (for further details see Methods). Data represents mean ± SD (n = 6). Statistical significance is indicated: effects of maternal hypoxia † *P*<0.05, †† *P*<0.01; effects of insulin *** *P*<0.001.

Pyruvate dehydrogenase (PDH), the rate-controlling step in glucose metabolism, was estimated as both the total amount of enzyme present, and that proportion possessing activity. MH increased total PDH activity in isolated hearts by 50% compared to fasted controls (*P*<0.01; Fig 4A). For control hearts active PDH represented ~10% of the total enzyme present (Fig 4B), whereas for MH-hearts this increased to 25% (*P*<0.01; Fig 4A). For control hearts, addition of insulin increased the active component of PDH 4-fold (*P*<0.001; Fig 4A), such that it now represented ~40% of the total PDH activity (*P*<0.001; Fig 4B), while for MH-hearts insulin did not affect the active component of PDH (NS; Fig 4A) and the active component remained at 25% of total PDH activity (NS; Fig 4B).

### Total metabolism

**Fig 5:**
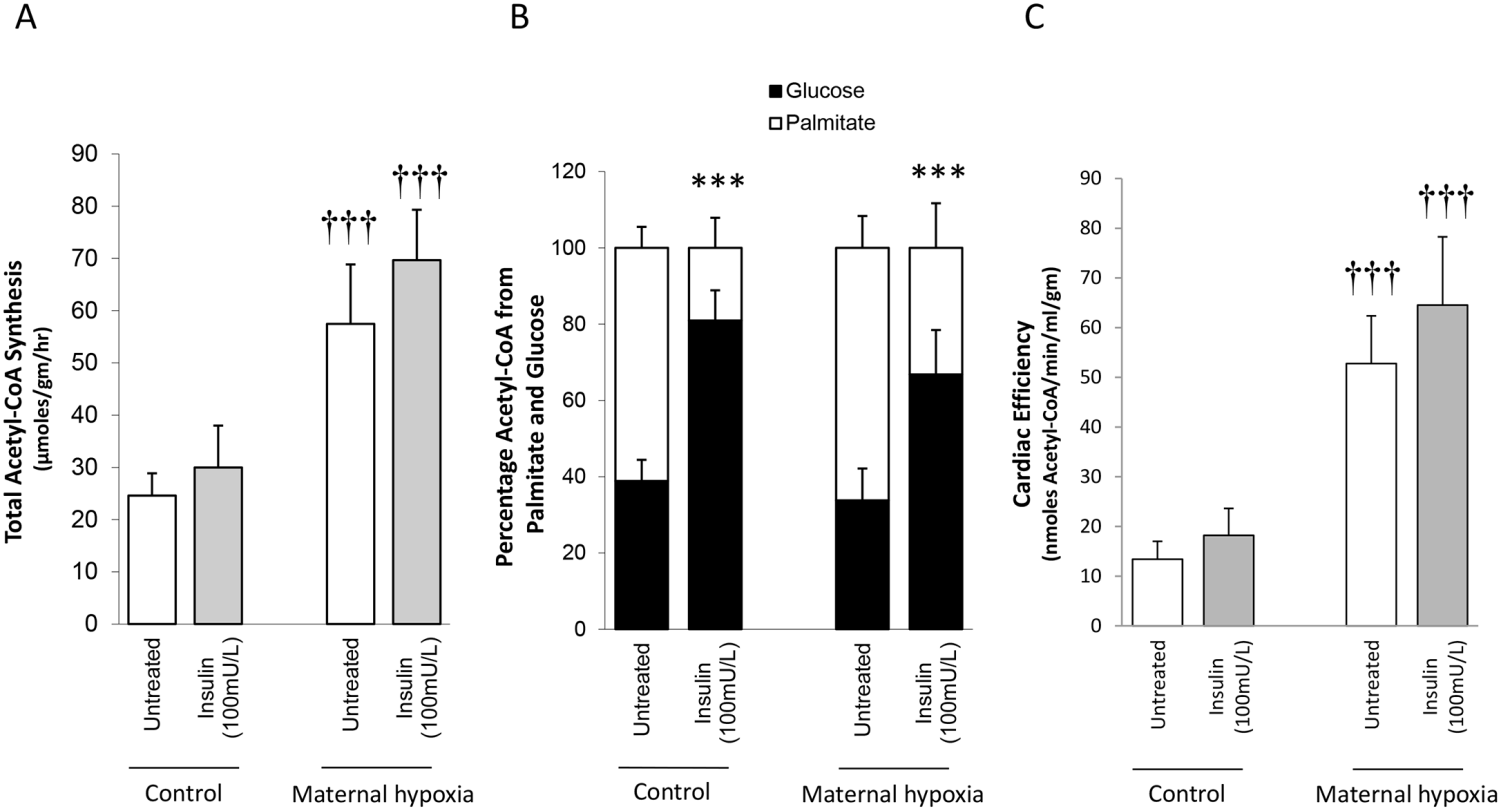
Estimates of total metabolism and metabolic efficiency for control and MH hearts. Total acetyl-CoA synthesis (A) was estimated from metabolism of glucose and palmitate, assuming that glucose yields 2 acetyl-CoA and palmitate yields 8 acetyl-CoA. Percentage of acetyl-CoA derived from each substrate (B) was estimated from absolute rates of metabolism. Cardiac efficiency (C) was calculated from total acetyl-CoA synthesis and cardiac output to estimate the metabolism per unit cardiac output. Data represents mean ± SD (n = 6). Statistical significance is indicated: effects of maternal hypoxia ††† *P*<0.001; effects of insulin *** *P*<0.001.

Estimates of total cardiac metabolism were calculated from rates of oxidation for glucose and palmitate. MH doubled the estimate of total metabolism calculated as acetyl-CoA production (*P*<0.001; Fig 5A), while for both fasted control- and MH-hearts 60% of the total acetyl-CoA was derived from palmitate (NS; Fig 5B). Following the addition of insulin, the proportion of acetyl-CoA derived from palmitate decreased to 20% for control hearts (*P*<0.001; Fig 5B), and for MH-hearts the effect was similar, with glucose providing 70% of the total acetyl-CoA (*P*<0.001; Fig 5B).

### Estimates of cardiac efficiency

Given that the majority of metabolic energy used by the heart is consumed to perform mechanical work, an estimate of efficiency was calculated to quantify the ‘cost’ per unit cardiac output. MH resulted in hearts that were less efficient, such that to perform cardiac work a 4-fold increase in acetyl-CoA production was needed compared with control hearts (*P*<0.001; Fig 5C). This was unaffected by the addition of insulin (NS; Fig 5C).

### Citrate synthase activity

**Fig 6:**
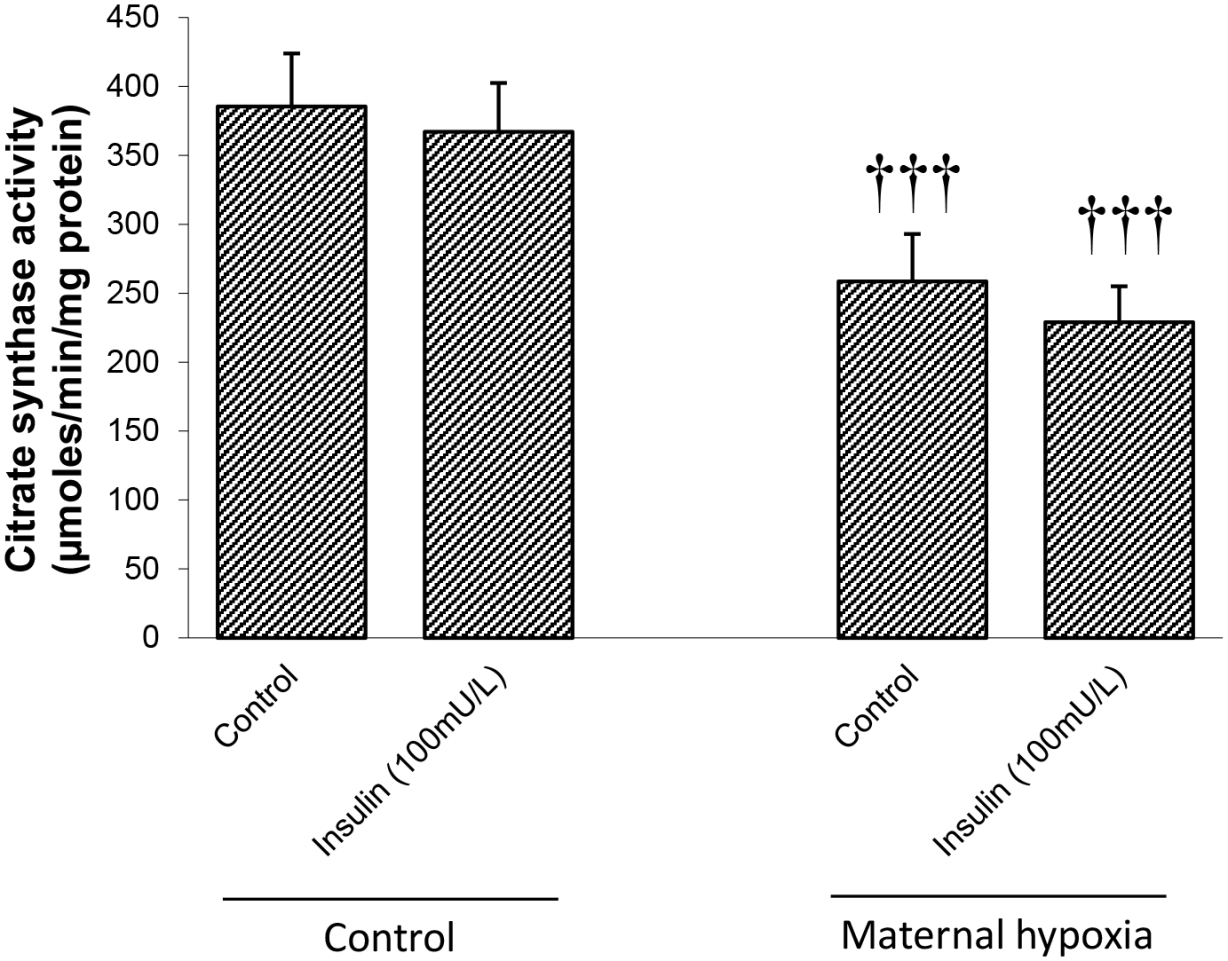
Effects of maternal hypoxia on absolute rates of citrate synthase activity in heart homogenates. Enzyme activity is presented with and without insulin stimulation. Data represents mean ± SD (n = 6). Statistical significance is indicated: effects of maternal hypoxia ††† *P*<0.001.

Total citrate synthase activity may be used as an index of cardiac mitochondrial content. MH led to a 30% decrease in citrate synthase activity when compared with control hearts (*P*<0.001; Fig 6), and was unaffected by insulin in untreated or MH hearts (NS; Fig 6).

### Theoretical oxygen kinetics

**Fig 7:**
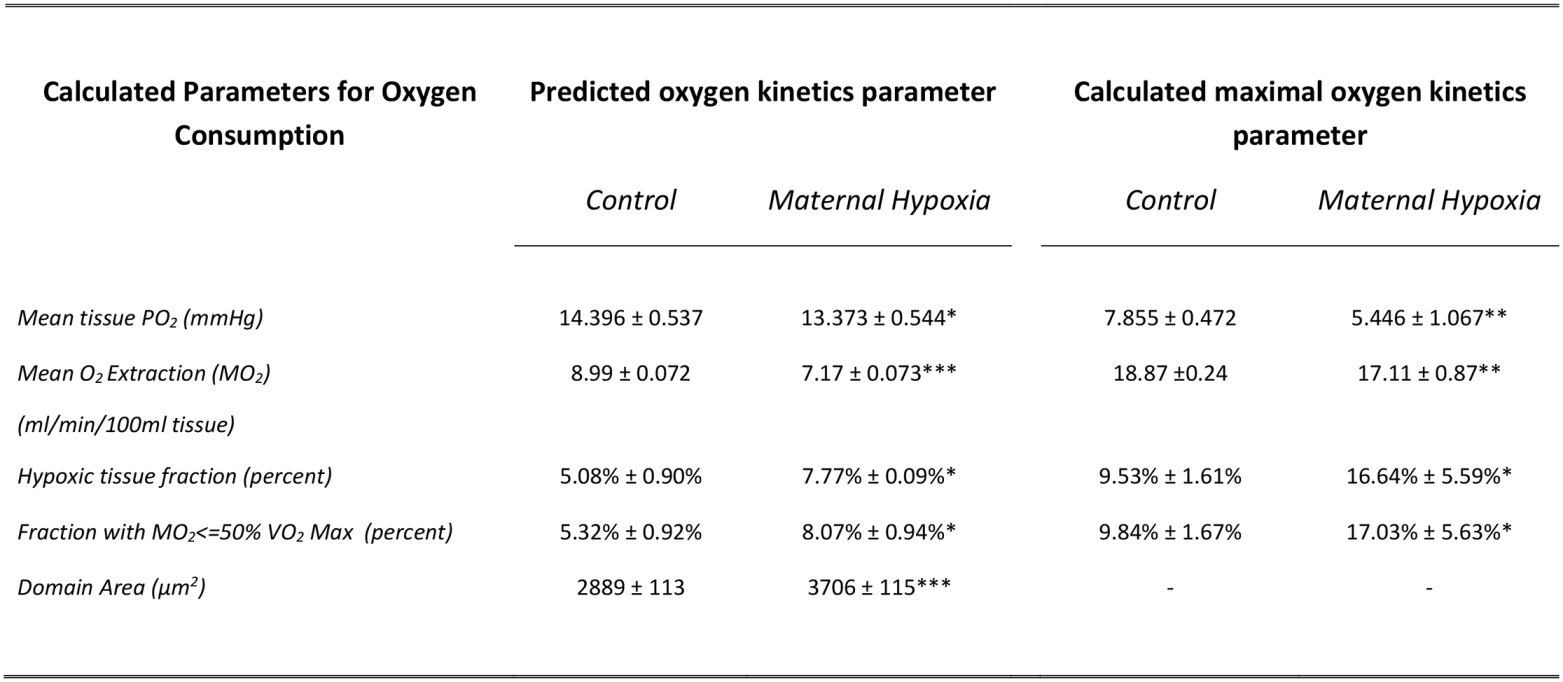
Predicted and maximal oxygen kinetics parameters for myocardium from control and MH rats. Maternal hypoxia was induced by exposing pregnant female rats to reduced oxygen tension (FIO_2_ = 0.12) for days E10-E20 of pregnancy. Offspring then developed up to 14weeks of age. Histological sections were stained and digitised to visualise capillaries as detailed in the methods section. Maximal oxygen kinetics were determined for maximal mitochondrial oxygen consumption predicting preserved mitochondrial density and capillary domain area as measured from the control and MH heart tissue. Data represents Mean ± SD (n = 6). Statistical significance represented as: effects of maternal hypoxia * P<0.05, ** P<0.01, *** P<0.001.

**Fig 8:**
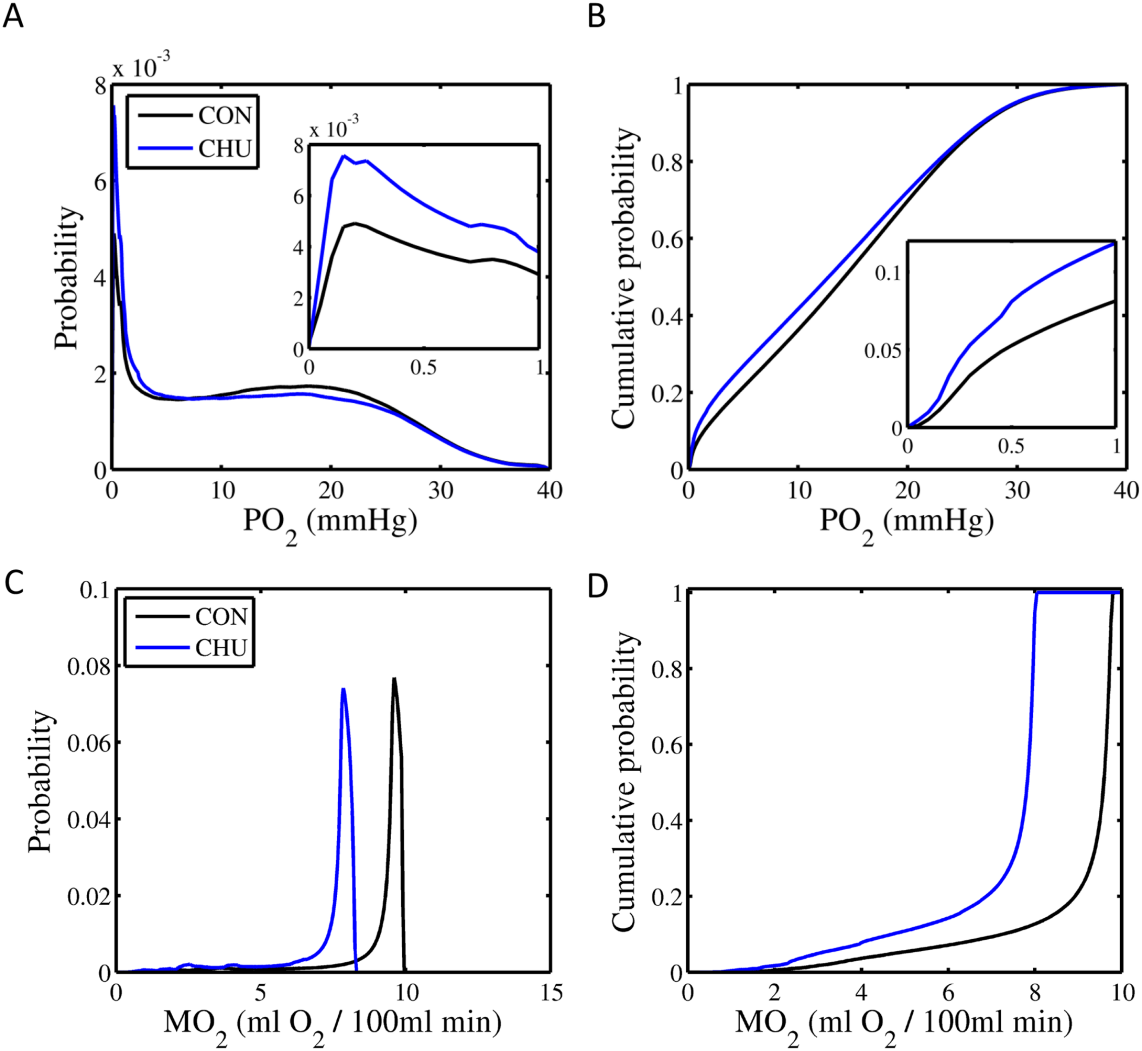
Calculated myocardial tissue oxygen partial pressure (PO_2_) and metabolic rate (MO_2_) for control and MH hearts. Probability (A) and cumulative probability (B) for tissue PO_2_ was calculated with reference to histological images to calculate capillary domain area, and citrate synthase activity to estimate the relative changes in tissue oxygen tension for control (CON) and hearts following chronic maternal hypoxia (CHU). Metabolic rate for individual myocytes (C) and cumulative probability (D) for metabolic rates were calculated from domain area and estimates of citrate synthase activity (for further details see Methods).

Based on digitised images, MH increased the calculated ventricular capillary domain area by 30% (*P*<0.05; Fig 7), and consequently the mean calculated tissue PO_2_ was decreased (8%; *P*<0.05; Fig 7) compared with control hearts. MH also decreased the calculated mean tissue oxygen extraction by 20% (*P*<0.001; Fig 7), and increased the tissue fraction categorised as hypoxic by 50% (*P*<0.05; Fig 7). This was accompanied by a 50% increase in the calculated region with tissue oxygen tensions indicative of < = 50% of cardiac tissue VO_2_max (*P*<0.05; Fig 7). Thus, modelling suggests that despite an increased domain area in MH hearts, the accompanying decreased oxygen consumption results in matching between calculated tissue PO_2_ for control and MH rats (Fig 8A). Indeed, estimates of cumulative PO_2_ indicate that only as PO_2_ declines to very low levels would a greater proportion of the myocardium achieve a PO_2_ corresponding to control tissue (Fig 8B). Measured decreases in citrate synthase activity was used to model reduced oxygen consumption in MH heart (Fig 8C), indicating that a greater proportion of the myocardium would likely have lower levels of oxygen consumption than in control hearts (Fig 8D).

To estimate the consequences of otherwise maintaining a normal mitochondrial density / oxygen consumption in hearts with an increased domain area, these calculations were repeated while simulating maximal oxygen consumption for both the control and MH geometries. The increased domain area in MH hearts (without the induced physiological responses) is predicted to lead to decreases in mean tissue PO_2_ of 30% (*P*<0.01; Fig 7), and a 10% decrease in mean tissue oxygen extraction (*P*<0.01; Fig 7). Consequently, hypoxic regions within the myocardium would increase by 75% for MH hearts (*P*<0.05; Fig 7), accompanied by a 75% increase in the area of myocardium with MO_2_< = 50% VO_2_max (*P*<0.05; Fig 7), without the observed changes in CS.

### Proteomic analysis

**Fig 9:**
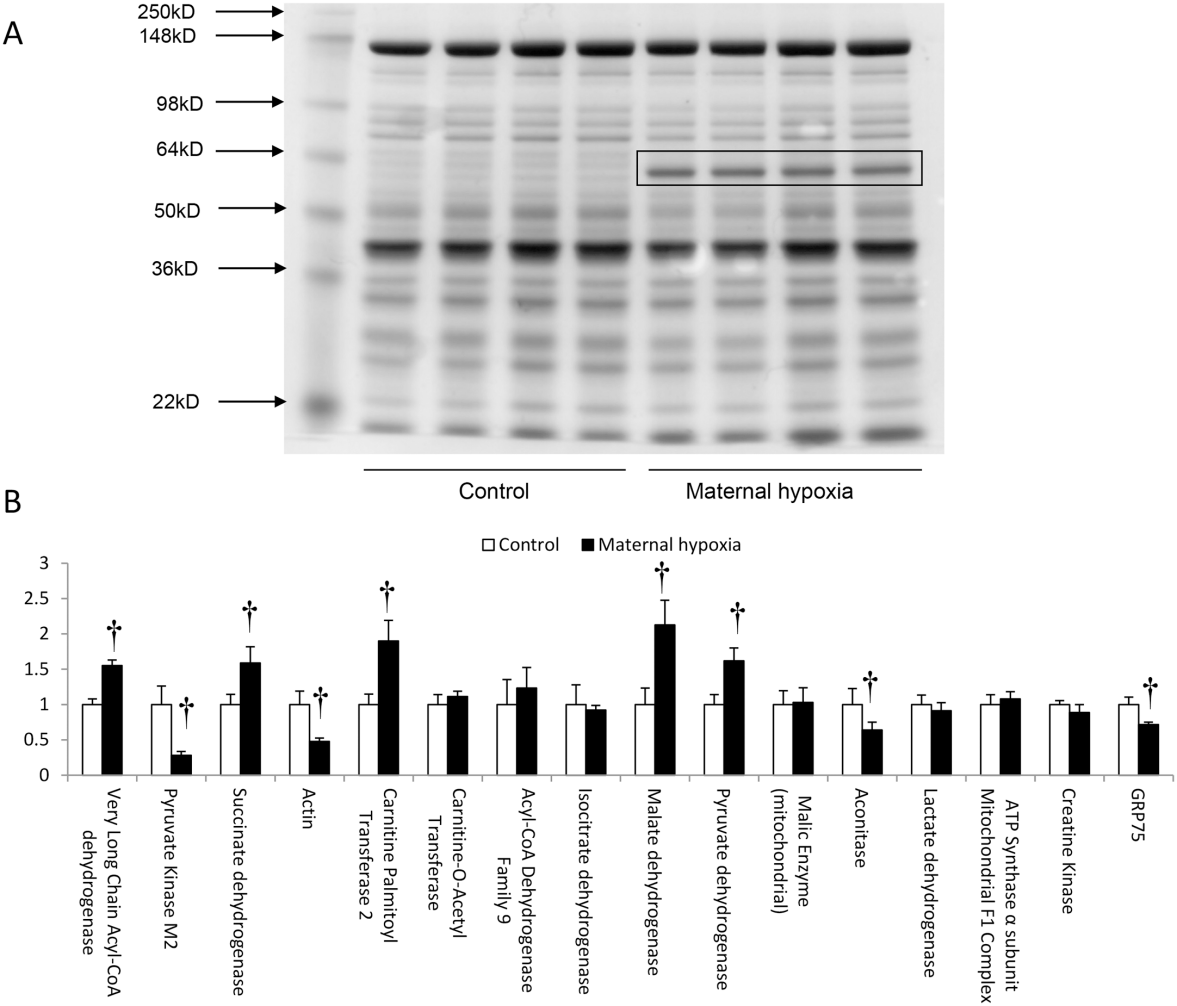
Changes to cardiac protein expression caused by MH in rats. (A) An example of reducing SDS polyacrylamide gel electrophoresis for control and MH heart tissue. (B) Proteomic analysis of the excised band corresponding to 78kd proteins isolated from gel, eluted from acrylamide and subjected to tryptic digestion before separation by HPLC and analysis by mass spectrometry (for further details see Methods). Data represents mean ± SD (n = 4). Statistical significance is indicated: effects of maternal hypoxia † *P*<0.05.

Separation of whole heart homogenates by reducing, SDS-polyacrylamide gel electrophoresis revealed a band of increased staining intensity in MH rats, corresponding to molecular weight 72.6 ± 1.4kd (n = 16; run on 4 separate gels; Fig 9A). Trypsinisation and analysis by LC-MS/MS revealed a range of protein fragments corresponding to proteins with 64kd-80kd molecular weight. Preliminary analysis revealed that proteins involved in fatty acid metabolism including very long-chain acyl-CoA dehydrogenase and carnitine palmitoyl-transferase 2 were increased (*P*<0.05 for both; Fig 9B), as were TCA cycle proteins including succinate dehydrogenase and malate dehydrogenase (*P*<0.05 for both; Fig 9B). In addition, pyruvate dehydrogenase protein fragments were increased (*P*<0.05; Fig 9B). By contrast, protein fragments corresponding to pyruvate kinase, aconitase, glucose regulated protein-75 (GRP75) and actin were decreased in abundance in MH-hearts (*P*<0.05 for all; Fig 9B).

## Discussion

These data represent the first demonstration that a more severe episode of MH than previously examined led to decreased cardiac output in the isolated perfused heart, suggesting that MH may alter a critical determinant for levels of physical activity, namely the potential to deliver oxygen to respiring tissues. Direct addition of insulin to the perfused heart had no effect on mechanical performance, indicating that the decrement in cardiac output was not a consequence of a mismatch in the provision of ATP from palmitate and glucose. Increasing efficiency of ATP production for the available oxygen by switching to predominantly glucose metabolism would increase ATP synthesis, and hence we must therefore reject the hypothesis that oxygen availability was the determining factor in altering cardiac output. Changes to performance may be a consequence of cardiac fibrosis, but the extent was very small and correlating degree of fibrosis with mechanical impairment is problematic as it is difficult to quantify the relative impact of the observed greater transverse and longitudinal collagen fibres to cardiac contractility. Changes to cardiac mechanics are also unlikely to result from altered myosin heavy chain subtypes, as previously noted by others [7]. Therefore, we speculate that the mechanical impairment in MH-hearts reported here was derived almost entirely from alterations to overall efficiency of metabolism, and hence ATP synthesis. Furthermore, these experiments highlight that the working heart preparation demonstrates decrements in cardiac performance that are absent in the Langendorff-perfused heart, which measures only the isometric contraction phase of cardiac function [4].

### Metabolism

Despite the decrease in mechanical performance, metabolism of substrates (glucose and palmitate) was increased by MH: glucose uptake doubled in the absence of insulin, suggesting the presence of increased glucose transporters in the plasma membrane. Adding insulin increased glucose metabolism in controls, indicating recruitment of GLUT4 to the cardiomyocyte surface from internal vesicular storage, but reciprocally decreased palmitate metabolism. For MH hearts insulin increased glucose uptake at similar magnitude to controls, suggesting there must still be vesicles containing recruitable GLUT4 present within the myocardium, and that the relative stimulus from insulin must be equivalent. Together, these observations imply preserved capacity within the insulin-signalling cascade involved in membrane transport of glucose for MH hearts. Insulin-increased glucose uptake and metabolism preserved the proportion of glucose (~5%) diverted through glycolysis to lactate in control hearts. Yet for MH-hearts, the proportion of glucose diverted to lactate decreased following insulin treatment, possibly as a consequence of end product inhibition of lactate dehydrogenase or the limited availability of cofactors such as NADH. Furthermore, the proportion of acetyl-CoA derived from both glucose and palmitate suggests that despite changes in absolute metabolism, the balance between glucose and palmitate energy provision was preserved. This supports the presence of a functioning Randle Cycle, giving rise to reciprocal regulation of fatty acid and carbohydrate metabolism, confirmed by direct *in vitro* measurement of unchanged cardiac ACC activity for both control and MH hearts (data not shown). Our results contrast with the only other investigation of metabolism in the MH heart [5], likely as a consequence of our prolonged exposure to MH and covering an earlier period in fetal development.

### Pyruvate dehydrogenase activity

Both palmitate and glucose produce the same intermediate (acetyl-CoA) that feeds directly into the TCA cycle. Entry of glucose into the TCA cycle is controlled by pyruvate dehydrogenase (PDH) in the outer mitochondrial membrane, thus preventing entry of glucose-derived acetyl-CoA during periods of limited glucose availability. Therefore, increased activity of PDH in MH hearts may be an important contributor to the greater glucose oxidation observed. For fasted control hearts active PDH forms a very small component of the total content, but while addition of insulin had no effect on total PDH it produced anticipated increases in the active component [24]. Insulin also increased the proportion of glucose stored in labelled and unlabelled glycogen pools, further demonstrating co-ordination between the path of glucose uptake and the fate of glucose for storage through glycogen synthase (GS), and the production of ATP.

By contrast, MH increased the total cardiac PDH and the active component above controls, in both absolute terms (enzyme activity measurements) and the proportion of active PDH. This observation was supported by proteomic analysis, likely through gene translation increasing levels of relevant functional proteins. With an increased contribution of glucose to total metabolism, at maximal levels of stimulation the potential flux through PDH is likely to be much higher following MH than in control hearts.

Surprisingly, addition of insulin to MH hearts had no net effect on the proportion of active PDH. Coupled with no change to the net rates of glycogen synthesis, this implies that either MH disrupted signalling between PDH/glycogen synthase and insulin, or the degree of stimulus provided by insulin was insufficient to further augment the activities of PDH and GS. Yet the augmented uptake for glucose was indicative of normally-coupled translocation of GLUT4 to the cardiomyocyte membrane, arguing that any interruption in insulin signalling is targetted. The putative decrease in expression of pyruvate kinase we note suggests a longer-term diversion of substrate away from anaplerotic reactions towards the synthesis of ATP.

### Cardiac efficiency

Our data suggests that the MH heart increased metabolism despite achieving lower cardiac output (less work), suggesting reduced intrinsic efficiency of the myocardium. When corrected for cardiac work MH increased the metabolic cost of cardiac output, implying either loss of metabolic energy or an increase in the internal work of the heart (mechanical resistance that needs to be overcome to facilitate contraction). Given the modest increases in fibrosis noted following MH, the latter cause appears unlikely. Indeed, failing hearts with significantly higher degrees of fibrosis show similar decreases in cardiac output despite modest reductions in overall mitochondrial metabolism [25]. Citrate synthase (CS) represents the rate-controlling step in TCA cycle [26], utilising the acetyl-CoA derived from glucose or palmitate, and has been used extensively as an index of mitochondrial density. Our data suggests that MH decreased CS activity by 30%, implying either decreases in relative mitochondrial volume or decreased cristae density of individual mitochondria. These data raise a paradox: MH increased total metabolism and hence mitochondrial function, yet total mitochondrial metabolic capacity was decreased. It is speculated that mitochondria possess a metabolic reserve, and under normal conditions are not functioning at maximal capacity to accommodate periods of metabolic stress [27]. Our data suggest that MH led to fewer mitochondria that all operated closer to the maximum capacity for metabolism, and hence will have a diminished scope to tolerate periods of metabolic stress as they are already utilising the functional reserve. Given that we measure a decrease in capillary density and associated reduction in diffusive exchange potential, coupled with a decrease in the total mitochondrial capacity per unit volume, how does this address an increase in the total metabolism measured? Using *in silico* experiments we have calculated oxygen consumption based on histological analysis and estimates of metabolic rate, coupled with the measured contributions from mitochondrial content.

### Oxygen kinetics modelling

We predicted that if capillary domain area increased in size and O_2_ consumption was maintained, then regions of hypoxia within individual myocytes would be larger in MH. The metabolic character of these cells is such that they would then not contribute to contractility through an inability to synthesise sufficient ATP. However, during systole these ‘non-contractile’ portions of the myocardium would need to be mobilised to produce adequate cardiac output, increasing internal work of the heart. Given that domain areas are increased 30% and CS activity declined 30% following MH, we speculate that those mitochondria are now effectively distributed over a larger domain area, thus diminishing the PO_2_ gradient between the capillary endothelium (highest PO_2_ region) to the midpoint between two adjacent capillaries—the Voronoi cell boundary (in essence the lowest PO_2_ region in the myocardium). Preserving mitochondrial density throughout a larger supply area would steepen the initial gradient for oxygen tension close to the capillary and increase the region of hypoxia within the fibres, potentially further increasing the internal work of contraction. In support of these speculations we recalculated oxygen tension, exploiting the maximum calculated oxygen consumption for both control and MH hearts using measured domain areas. We demonstrate that preserving normal mitochondrial density and hence oxygen consumption at higher levels in MH would significantly increase the regions of hypoxia within the calculated domain area and, more importantly, drastically increase the area where PO_2_ supports < = 50% VO_2_max, the region over which metabolism must switch between fatty acid and glucose in order to maximise ATP synthesis. Indeed, heterogeneity in mitochondrial distribution within skeletal muscle may be dictated by capillary supply (and hence oxygen delivery) in both the mouse and non-mammalian species [28]. Our calculations of O_2_ tension in MH hearts imply that decreased CS activity (and by inference oxygen consumption) may be necessary to maximise metabolism within individual capillary supply areas, thus preserving the greatest proportion of contractile myocardium. One shortcoming associated with this mechanism may be the inability to mobilise further residual metabolic capacity within the mitochondria during periods of metabolic stress, as there may be insufficient oxygen available to meet the ATP production required. This would subsequently reduce exercise intolerance, an important indicator of overall health.

### Experimental limitations

Our observations are made with a high partial pressure, low-oxygen content perfusion medium [29] at very high coronary flow rates. For the crystalloid-perfused heart arteriole dilatation is anticipated to be maximal, as this overcomes autoregulation of coronary arterioles by perfusing with high O_2_ partial pressure yet low O_2_ content perfusate [29]. Oxygen delivery for the blood-perfused heart may be less sensitive to modest changes to coronary flow [30], despite lower flow rates, as supply is uncoupled from flow rate [31]. Previous experiments postulate diffusion of oxygen from other elements of the vasculature (arterioles) [32], however given the very high flow rates in perfused hearts coupled with the short length of arterioles, we predict that diffusion will be almost entirely through the capillary network [33].

The oxygen diffusion modelling was undertaken using images from previously-perfused hearts and we therefore cannot exclude changes to the architecture of the heart by tissue oedema. However, care was taken to minimise this effect by using untreated samples that had been perfused for the same (short) duration and are therefore representative of the experiment.

We are justified in exploiting hypoxia to manipulate maternal arterial blood gas concentrations as we demonstrate that hypoxia led to a modest relative hypocapnia but no change to either bicarbonate concentration or blood pH.

Given that MH rats develop hypertension in later life (Dr. W. Rook, University of Birmingham, personal communication) the reduction in capillary density at 14weeks [4] may be part of the later development of an overt hypertension [34].

## Conclusions

We demonstrate that a prolonged period of MH decreases cardiac output, and hence has the potential to decrease the exercise capacity of an individual. This occurred despite an apparent increase in cardiac metabolism of glucose and fatty acids, suggesting that mitochondria were inefficient, possibly as a consequence of proton leakage/uncoupling or oxidative stress. In order to accommodate subsequently altered oxygen kinetics decreasing mitochondrial density within a capillary supply area may, paradoxically, improve the contractile activity of individual muscle fibres by decreasing the size of hypoxic regions within myocytes. This adaptive change may facilitate preservation of cardiac output despite metabolic inefficiency, but possibly increases sensitivity of the myocardium to further metabolic stresses. Our study highlights potential targets for new experiments exploiting full genomic and proteomic analysis, in addition to investigation of mitochondrial integrity that were beyond the scope of the original study. In addition, we will in future studies examine insulin signalling to investigate the factors effecting PDH/PDK and glycogen synthase regulation.

## Supporting Information

S1 Fig Supplemental Material. (DOCX)

S2 Fig Accession numbers identifying protein fragments of interest identified by mass spectrometry from proteomic analysis of electrophoresis gel fragment. For further details, see methods section.
(DOCX)

S3 Fig Estimates of tissue fibrosis from picro-sirius stained left ventricle. Collagen infiltration was estimated from a point-counting method to determine percentage of area corresponding to fibrosis. For further details see methods. Data represents Mean ± SD (n = 6 hearts). Statistical significance indicated * P<0.05.
(PPTX)

S4 Fig Estimates of capillary from alkaline phosphatase stained left ventricle. Capillary density was estimated from counting of stained points corresponding to capillaries in area of defined size. For further details see methods. Data represents Mean ± SD (n = 6 hearts). Statistical significance indicated ** P<0.01.
(PPTX)

S5 Fig Estimates of tissue myosin heavy chain protein isoforms for left ventricle from control and MH rat hearts. Homogenates were isolated from left ventricle in a high PO_4_^=^ containing buffer and supernatants recovered. Samples were diluted using a non-denaturing, non-reducing sample buffer and loaded onto a polyacrylamide (6% w/v) gel containing glycerol (45% w/v) with 20mM pyrophosphate. Each sample corresponded to 0.5mg ventricle protein. Gels were run for 24hr at 4°C before staining with Coomassie Brilliant Blue Stain. For further details see Garcia et al. 2007 Eur. J. Physiol. 454 p.937–943. Densitometry was determined using computer software (ImageJ, NIH). Data represents Mean ± SD (n = 6).
(PPTX)

S6 Fig Calculated myocardial tissue oxygen partial pressure (PO_2_) and metabolic rate (MO_2_) for control (CON) and Chronic maternal hypoxic (CHU) hearts using maximum metabolic rates estimates to determine the effects of normal mitochondrial density coupled with larger domain area. Probability (A) and cumulative probability (B) for tissue PO_2_ was calculated with reference to histological images to calculate domain area and citrate synthase activity to estimate the relative changes in tissue oxygen tension following CHU. Metabolic rate for individual cells (C) and cumulative probability (D) for metabolic rates were calculated from domain area and maximum predicted oxygen extraction measurements were used. For further details see methods.
(PPTX)

S7 Fig Arterial blood gases for anaesthetised pregnant rats (Gestational Day = 13–15) breathing room air or 12% oxygen. For further details see S1 Fig. Data represents mean ± SD (n = 6). Statistical significance represented: effects of hypoxia * P<0.05, ***P<0.001 (Paired students‘t’ test). Arterial blood gases were measured in pregnant rats to determine the effects of hypoxia (FIO_2_ = 0.12) on blood gases during pregnancy and, by extension, on the foetal environment. We note no changes to either pH or bicarbonate concentration in arterial plasma, with the greatest effect demonstrated as arterial hypoxaemia. The data indicate that we are justified in exploiting FIO_2_ as a mechanism to fix oxygen concentration without subsequent changes to plasma PaCO_2_, pH or bicarbonate concentrations. We demonstrate that anaesthesia has undoubtedly blunted arterial oxygen tension and led to relative hypercapnia compared with other evidence of hypocapnia as a consequence of pregnancy in humans [Moore et al. 1987 J. Appl. Physiol. 62 p.158–163, Lueder et al. 1995 Metabolism 44 p.532–537] and rats [Lueder et al. 1995 Metabolism 44 p.532–537].
(DOCX)
