# Supplementary material for: Maternal Hypoxia Decreases Capillary Supply and Increases Metabolic Inefficiency Leading to Divergence in Myocardial Oxygen Supply and Demand
Source: PLoS One. 2015 Jun 1;10(6):e0127424. doi: 10.1371/journal.pone.0127424 (PMC4452690; doi:10.1371/journal.pone.0127424)
Supplement: S1 Fig — (DOCX) [file pone.0127424.s001.docx]

**Measurement of blood gas partial pressure**

To measure arterial blood gases from anaesthetised rats, animals were prepared surgically as detailed below. Briefly, pregnant female rats (13-15days post-fertilisation; 3-5days post transfer to hypoxia FIO_2_=0.12) were initially anaesthetised with isoflurane (3-4 % in oxygen) whilst the left jugular vein was cannulated, then α-chloralose and urethane (165 mg and 2.5 g in 10 ml saline i.v.) infused. Depth of anaesthesia was monitored by pedal reflex response. A tracheal cannula was used to allow spontaneous breathing and attach a spirometer. The left femoral artery was cannulated to measure arterial blood pressure and blood gases (GEM4000, Instrumentation Laboratory, Warrington, Cheshire, UK). Following recording of baseline parameters breathing room air, rats were exposed to hypoxia (12% oxygen—balance nitrogen) and arterial oxygen, carbon dioxide, bicarbonate and pH recorded at steady state. Gas mixtures were combined by rotameter from sources of oxygen and nitrogen.

**Citrate synthase activity**

Citrate synthase activity was measured by the method of Morgunov and Srere [1]. Briefly, cardiac tissue samples were homogenised in homogenisation buffer (Tris.HCl, 0.1M final concentration; EDTA, 1mM final concentration; 0.25% (v/v) Triton X-100; adjusted to pH=8.0). Samples were centrifuged at 1,000g for 2min and stored on ice. Activity was measured using a thermostatically-controlled heated cell (37°C) using an assay buffer comprising Tris.HCl (0.1M); EDTA (1mM); Triton X-100 (0.25%v/v), DTNB (0.1mM) and acetyl-CoA (0.31mM). Typical sample volumes used 10-20µl. Assays were pre-incubated for 5min and were started by addition of oxaloacetate (0.5mM final concentration) and activity measured at 412nm. Rates of reaction were expressed relative to homogenate protein content. Protein concentrations were estimated using BCA protein assay (Sigma, Poole, UK).

**Acetyl-CoA carboxylase (ACC) activity**

Cardiac ACC activity was estimated by the bicarbonate fixation method of Saddik *et al.* [2]. Briefly, tissues were homogenised in buffer containing Tris-HCl (50 mM, pH=7.5), NaF (100 mM), EDTA (2 mM), sucrose (0.25 M), and mercaptoethanol (70 μl/100 ml). Homogenates were centrifuged (18 000 rpm, 30 minutes); and the supernatant was dialyzed against buffer containing Tris-HCl (50 mM, pH=7.5), NaF (100 mM), EDTA (2 mM), mercaptoethanol (10 mM), and glycerol (10% v/v). The ACC activity was quantified in dialysate protein (100 μg) added to reaction mixture (final volume, 190μL) containing Tris-HCl (11.5 mM, pH = 7.5), bovine albumin (2.9 μM), mercaptoethanol (1.5 μM), ATP (0.41 mM), acetyl-CoA (0.21 mM), magnesium acetate (0.97 mM), and NaHCO_3_ (3.5 mM) supplemented with ^14^C-labeled NaHCO_3_ (final reaction, 1.375 μCi/ml). Reactions were incubated for 5 minutes at 37°C and terminated by addition of 6% (w/v) perchloric acid (200 μl). Radioactivity was estimated in the aqueous fraction by liquid scintillation counting. Homogenate protein concentrations were estimated using BCA protein assay (Sigma, Poole, UK).

**Pyruvate dehydrogenase activity**

Pyruvate dehydrogenase activity was measured using the method of Seymour and Chatham [3]. Briefly, two components of pyruvate dehydrogenase (PDH) activity were isolated, total and active:

**Total PDH activity**

Enzyme activity was extracted from homogenised tissue using buffer comprising of: HEPES (75mM), dichloroacetate (5mM), MgCl_2_ (5mM), ADP (1mM), dithiothreitol (0.05mM) and Triton X-100 (1%) adjusted to pH=7.0. Tissue was homogenised using a blade homogeniser (Ultraturrax) and centrifuged (15,000g, 10min) and the supernatant recovered.

**Active PDH activity**

Enzyme activity was extracted from homogenised tissue using buffer comprising of: HEPES (25mM), KH_2_PO_4_ (25mM), KF (25mM), dichloroacetate (1mM), EDTA (3mM), ADP (1mM), dithiothreitol (1mM) and Triton X-100 (1%) adjusted to pH=7.0. Tissue was homogenised using a blade homogeniser (Ultraturrax) and centrifuged (15,000g, 10min) and the supernatant recovered.

**Assay conditions**

Assay buffer comprising HEPES (50mM), MgCl2 (1mM), EGTA (0.08mM), DTT (1mM), rotenone (4µM), NAD (1.67mM), Co-enzyme A (0.1mM), thiamine pyrophosphate (0.2m M), lactate (16.7mM) and lactate dehydrogenase (2U) adjusted to pH=7.2 were incubated at 30°C. Sample was added and the progress of the reaction was followed at 340nm. Enzyme activity was calculated with reference to homogenate protein concentrations, estimated using BCA protein assay (Sigma, Poole, UK).

**Oxygen diffusion calculations**

Binary images of total capillary supply for identical regions were taken (above). The shortest distance between adjacent capillaries was calculated by Delaunay triangulation, producing a value for the summed influence of all nearest neighbours [4], and giving an assessment of the variance in capillary supply in the normal state and following MH. The capillary supply area was calculated following Voronoi tessellation of digitised images, to produce non-overlapping polygons centred around an individual vessel (capillary domains), whose boundaries were defined as a point equidistant between two adjacent capillaries, i.e. representing the half-maximal distance between ‘nearest neighbours’ [5]. Coefficient of variation was calculated for capillary density, domain area, and nearest neighbour distance (NN) as standard deviation/mean x100.

PO_2_ heat maps were generated using a two-dimensional circular region within a cross section of heart muscle tissue. This was modelled as a homogeneous tissue composition, with an array of capillaries of circular cross-section (with radius, *r_cap_* = 1.8 *µ*m) supplying O_2_ to the tissue with Michaelis-Menten consumption kinetics; O_2_ transport within the myocardium was therefore described by the equation

$$\alpha D\nabla^{2}p-\frac{M_{0}p}{p+p_{c}}=0, (1)$$

where *p* is tissue PO_2_, α is tissue O_2_ solubility, *D* is tissue O_2_ diffusivity, *M_0_* is the maximum tissue O_2_ demand, and *p_c_* is tissue PO_2_ when O_2_ consumption is half of the demand. At each capillary-tissue interface, the flux is proportional to the difference in PO_2_ across this wall, and was thus described by the boundary condition

$$n\cdot\left[ \alpha D\nabla p \right]-k\left( p_{cap}-p \right)=0, (2)$$

where *p_cap_* is the transversally averaged intracapillary PO_2_, *k* is the mass transfer coefficient, and *n* denotes the outward unit normal*.* Also, at the exterior boundary a no-flux condition was assumed

$$n\cdot\left[ \alpha D\nabla p \right]=0, (3)$$

though sufficiently far into the sample area, the solution is insensitive to the details of this boundary condition [6]. Equations (1-3) were solved to determine tissue PO_2_ using the finite element method, implemented using Matlab (The MathWorks Inc., Natick, MA) with a nonlinear solver based on Gauss-Newton iteration and adaptive meshing to resolve areas of rapid change in oxygen partial pressures.

In the simulations, we used a capillary density of 1400 capillaries per mm^2^, which fixes the physical domain size for a given number of capillaries, and we also use:

*α* = 3.89e-5 mL O_2_ mL^-1^ mmHg^-1^, *D* = 2.41e-5 cm^2^ s^-1^, *p_cap_* = 20 mmHg
*k =* 4.0e-6 mL O2 s-1 cm-2 mmHg^-1^, *M_0_* = 1.57e-4 mL O_2_ mL^-1^ s^-1^, *r_cap_* = 1.8 *µ*m,

as suggested by [7]. In addition, we take use p_c_ = 0.5 mmHg, based on estimates presented in Wilson et. al [8].

**Proteomics analysis**

**Gel electrophoresis**

Briefly, cardiac tissue (50 mg) was powdered in liquid nitrogen and extracted with radioimmunoprecipitation assay (RIPA) buffer containing protease and phosphatase inhibitors, followed by centrifugation (10 000 rpm for 10mins) and recovery of the supernatant. Samples were diluted with 5x sample buffer (containing mercaptoethanol as reducing agent) to give a final protein concentration 2mg/ml. Samples (40µg) were loaded onto a reducing SDS-PAGE gel (10% w/v bis-acrylamide). Gels were run at constant current (~100mA) and the resolved gel recovered and stained with Coomassie Brilliant Blue protein stain. Bands were identified visually using a lightbox and regions of interest were excised from the gel with a sterile scalpel. Fragment molecular weight were determined with regard to a pre-stained molecular weight ladder (SeeBlue Plus 2, Invitrogen) and estimated from relative distance travelled across the gel using computer software (Image J, NIH).

**Sample trypsinisation**

Coomassie bands were excised, divided (~2 mm^3^ cubes) and de-stained with acetonitrile followed by ammonium bicarbonate (100mM). De-stained gel pieces were dried (vacuum centrifugation; 5 min) and rehydrated in DTT (10mM), ammonium bicarbonate (100mM). Gel pieces were reduced at 60°C for 15 min, the liquid was removed and replaced with iodoacetamide (50mM), ammonium bicarbonate (100mM). Gel pieces were incubated at room temperature in the dark for 45 min and then washed with ammonium bicarbonate (100mM). De-stained gel pieces were dried (vacuum centrifugation; 5 min) and 20µg trypsin gold added (Promega, WI, USA), shaken at room temperature for 30 min, before dilution with ammonium bicarbonate (100mM). Hydrolysis was allowed to occur overnight (~16 h) at 37 °C. Peptides were extracted with the initial solution of 2% (w/v) acetonitrile, 0.1% (w/v) formic acid in water was added and shaken for 30 minutes. Supernatant was removed to a clean tube. A second peptide extraction was performed using 40% (w/v) acetonitrile, 0.1% (w/v) formic Acid in water, shaken for 30 minutes at room temperature. The supernatant was removed, pooled with the previous extracted peptides and dried in an evaporator. The samples were re-suspended in 0.1% (w/v) formic acid/water in preparation for the mass spectrometry analysis.

**Mass spectrometry analysis**

UltiMate^®^ 3000 HPLC series (Dionex, Sunnyvale, CA USA) was used for peptide concentration and separation. Samples were separated in Nano Series™ Standard Columns (75 µm i.d. x 15 cm) packed with C18 PepMap100 (3 µm, 100Å). The gradient used was from 3.2% to 44% solvent B (0.1% formic acid in acetonitrile) for 30 min. Peptides were eluted directly (~ 350 nL min^-1^) *via* a Triversa Nanomate nanospray source (Advion Biosciences, NY) into a LTQ Orbitrap Velos ETD mass spectrometer (ThermoFisher Scientific, Germany). The data-dependent scanning acquisition was controlled by Xcalibur 2.1 software. The mass spectrometer alternated between a full FT-MS scan (m/z 380 – 1600) and subsequent collision-induced dissociation (CID) MS/MS scans of the 7 most abundant ions. Survey scans were acquired in the Orbitrap with a resolution of 60,000 at m/z 400 and automatic gain control (AGC) 1x10^6^. Precursor ions were isolated and subjected to CID in the linear ion trap with AGC 1x10^5^. Collision activation for the experiment was performed in the linear trap using helium gas at normalized collision energy to precursor m/z of 35% and activation Q 0.25. The width of the precursor isolation window was 2 m/z and only multiply-charged precursor ions were selected for MS/MS. The MS and MS/MS scans were searched against NCBInr database using Mascot algorithm (Matrix Sciences) and software Proteome Discoverer 1.3 (ThermoFisher Scientific, Germany) to identify candidate peptides (Figure S2). Variable modifications were deamidation (N and Q), oxidation (M) and phosphorylation (S, T and Y). The precursor mass tolerance was 10 ppm and the MS/MS mass tolerance was 0.8Da. Two missed cleavage was allowed and were accepted as a real hit protein with at least two high confidence peptides.

**References**

[1] Morgunov I, and Srere PA (1998) Interaction between citrate synthase and malate dehydrogenase’ J. Biol. Chem. 273: 9540-29544.

[2] Saddik M, Gamble J, Witters LA, Lopaschuk GD (1993) Acetyl-CoA carboxylase regulation of fatty acid oxidation in the heart. J Biol. Chem. 268: 25836–25845.

[3] Seymour AM, Chatham JC (1997) The effects of hypertrophy and diabetes on cardiac pyruvate dehydrogenase activity. J. Mol. Cell. Cardiol. 29: 2771-2778.

[4] Egginton S, Ross HF (1989) Influence of muscle phenotype on local capillary supply. Adv. Exp. Med. Biol. 247: 281-291.

[5] Karch R, Neumann RF, Ulrich R, Neumüller RJ, Podesser BK, et al. (2005) The spatial pattern of coronary capillaries in patients with dilated, ischemic or inflammatory cardiomyopathy. Cardiovasc. Path. 14: 135-144.

[6] Al-Shammari AA, Gaffney EA, Egginton S (2012) Re-evaluating the Use of Voronoi Tessellations in the Assessment of Oxygen Supply from Capillaries in Muscle. Bull. Math. Bio. 74: 2204-31.

[7] Beard DA, Schenkman K, Feigl O (2003) Myocardial oxygenation in isolated hearts predicted by an anatomically realistic microvascular transport model. Am. J. Physiol. 285: H1826-H1836.

[8] Wilson DF, Rumsey WL, Green TJ, Vanderkooi JM (1988) The oxygen dependence of mitochondrial oxidative phosphorylation measured by a new optical method for measuring oxygen concentration. J. Biol. Chem. 263: 2712-2718.
