# Supplementary material for: Maternal Hypoxia Decreases Capillary Supply and Increases Metabolic Inefficiency Leading to Divergence in Myocardial Oxygen Supply and Demand
Source: PLoS One. 2015 Jun 1;10(6):e0127424. doi: 10.1371/journal.pone.0127424 (PMC4452690; doi:10.1371/journal.pone.0127424)
Supplement: S2 Fig — For further details, see methods section. (DOCX) [file pone.0127424.s002.docx]

| Peptide Accession Number | Protein Fragment Identification |
| --- | --- |
| 1000439 | Glucose-Regulated Protein 75 (grp75 – mitochondrial HSP70 Chaperone family) [Rattus sp.] |
| 10637996 | mitochondrial aconitase [Rattus norvegicus] |
| 149018984 | malic enzyme 1 [Rattus norvegicus] |
| 149029483 | ATP synthase, H+ transporting, mitochondrial F1 complex, alpha subunit, isoform 1, isoform CRA_d [Rattus norvegicus] |
| 149048722 | acyl-Coenzyme A dehydrogenase family, member 9, isoform CRA_c [Rattus norvegicus] |
| 158341689 | NADP-dependent malic enzyme [Rattus norvegicus] |
| 18426858 | succinate dehydrogenase [ubiquinone] flavoprotein subunit, mitochondrial precursor [Rattus norvegicus] |
| 1850592 | carnitine palmitoyltransferase II [Rattus norvegicus] |
| 4885049 | actin, alpha cardiac muscle 1 proprotein [Rattus norvegicus] |
| 51854229 | carnitine O-acetyltransferase [Rattus norvegicus] |
| 56541110 | Acyl-Coenzyme A dehydrogenase, very long chain [Rattus norvegicus] |
| 62079055 | isocitrate dehydrogenase [NADP], mitochondrial precursor [Rattus norvegicus] |
| 6671762 | creatine kinase M-type [Mus musculus] |
| 6981146 | L-lactate dehydrogenase B chain [Rattus norvegicus] |
| 78365255 | dihydrolipoyllysine-residue acetyltransferase component of pyruvate dehydrogenase complex, mitochondrial [Rattus norvegicus] |
| 89574117 | mitochondrial malate dehydrogenase 2, NAD [Rattus norvegicus] |
