# Supplementary figures and images for: Maternal Hypoxia Decreases Capillary Supply and Increases Metabolic Inefficiency Leading to Divergence in Myocardial Oxygen Supply and Demand
Source: PLoS One. 2015 Jun 1;10(6):e0127424. doi: 10.1371/journal.pone.0127424 (PMC4452690; doi:10.1371/journal.pone.0127424)

## Slide 1
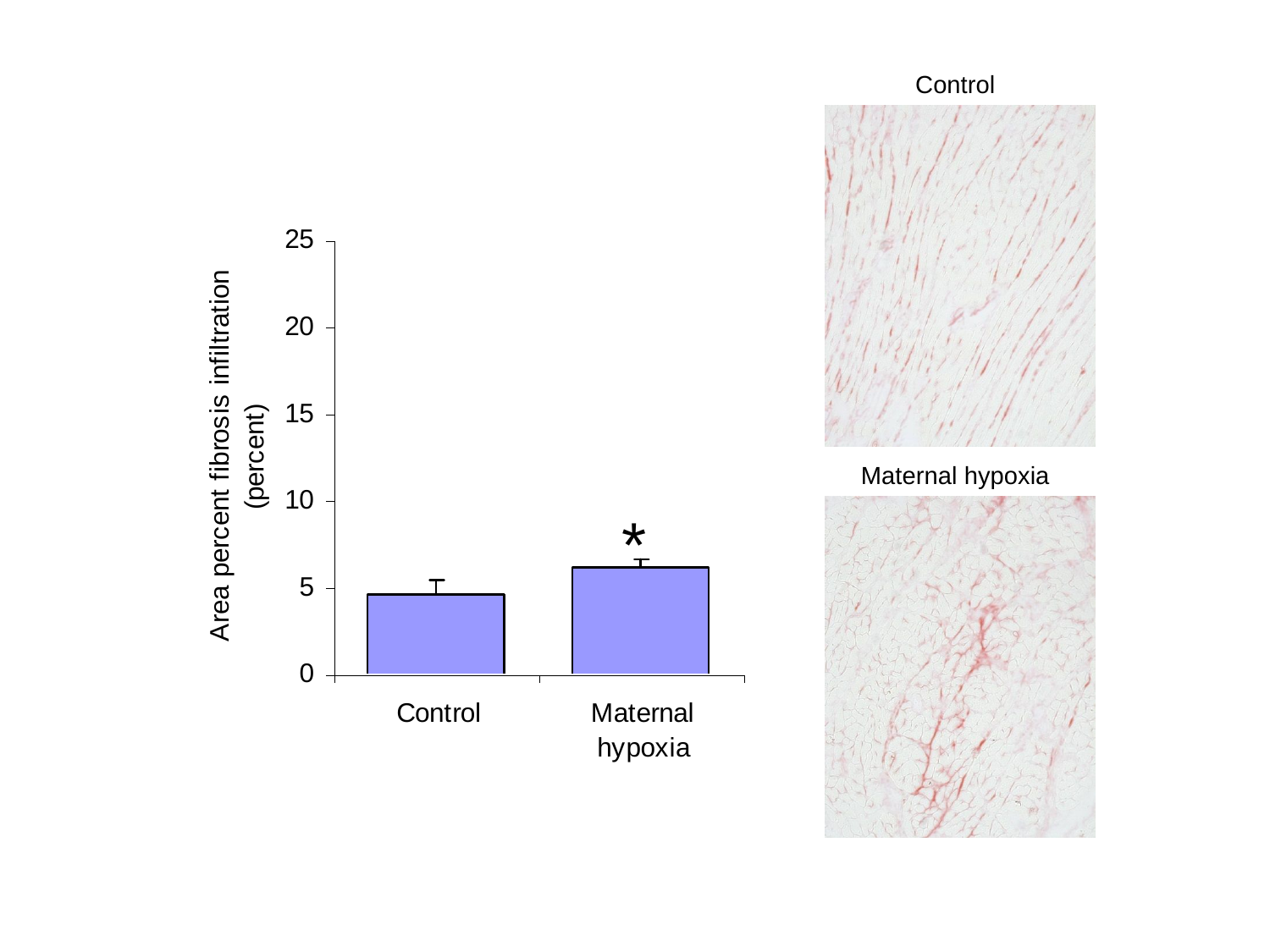

Control
Maternal hypoxia
*

Supplement: S3 Fig — Collagen infiltration was estimated from a point-counting method to determine percentage of area corresponding to fibrosis. For further details see methods. Data represents Mean ± SD (n = 6 hearts). Statistical significance indicated * P<0.05. (PPTX) [file pone.0127424.s003.pptx]

## Slide 1
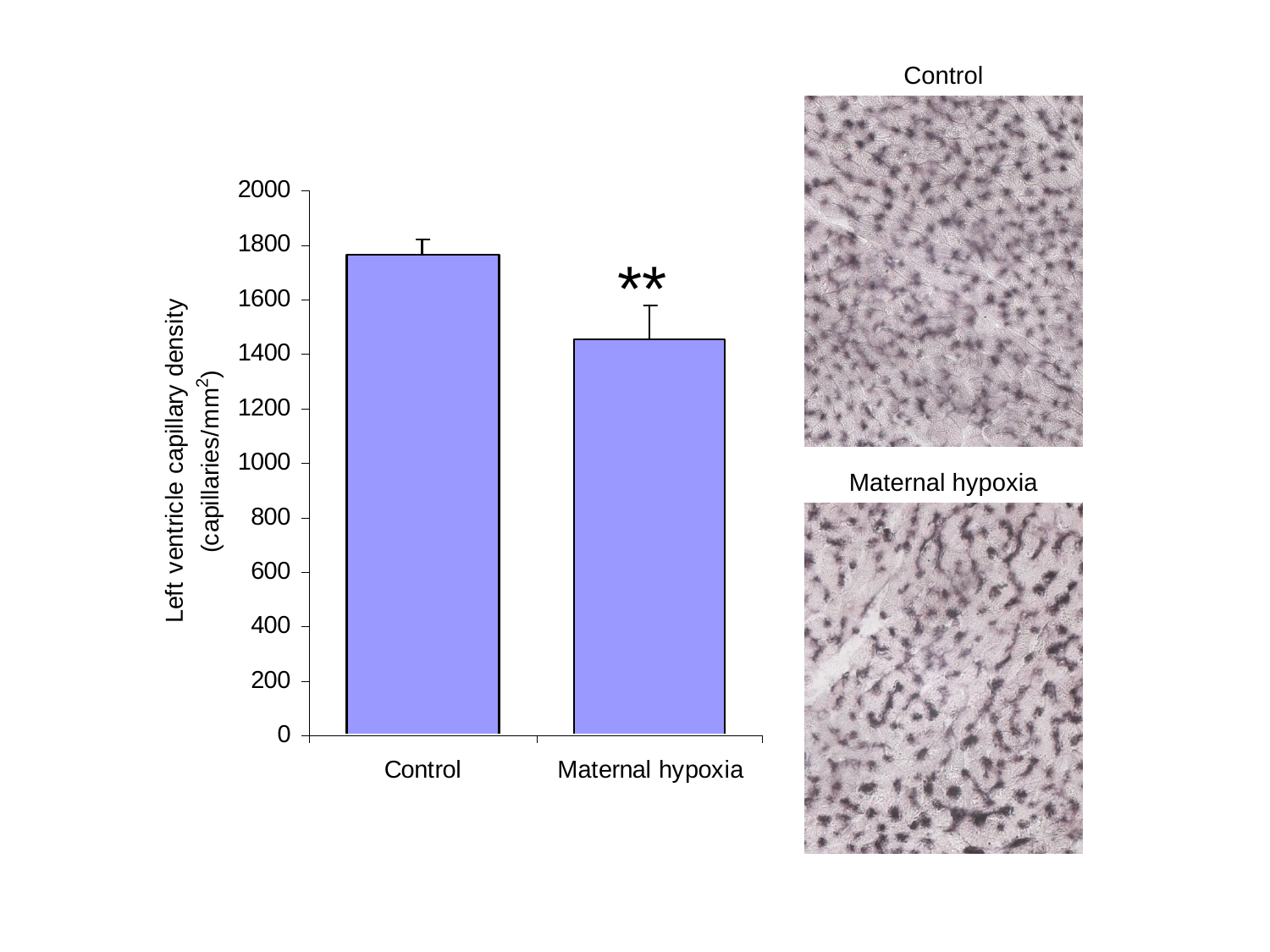

Control
**
Maternal hypoxia

Supplement: S4 Fig — Capillary density was estimated from counting of stained points corresponding to capillaries in area of defined size. For further details see methods. Data represents Mean ± SD (n = 6 hearts). Statistical significance indicated ** P<0.01. (PPTX) [file pone.0127424.s004.pptx]

## Slide 1
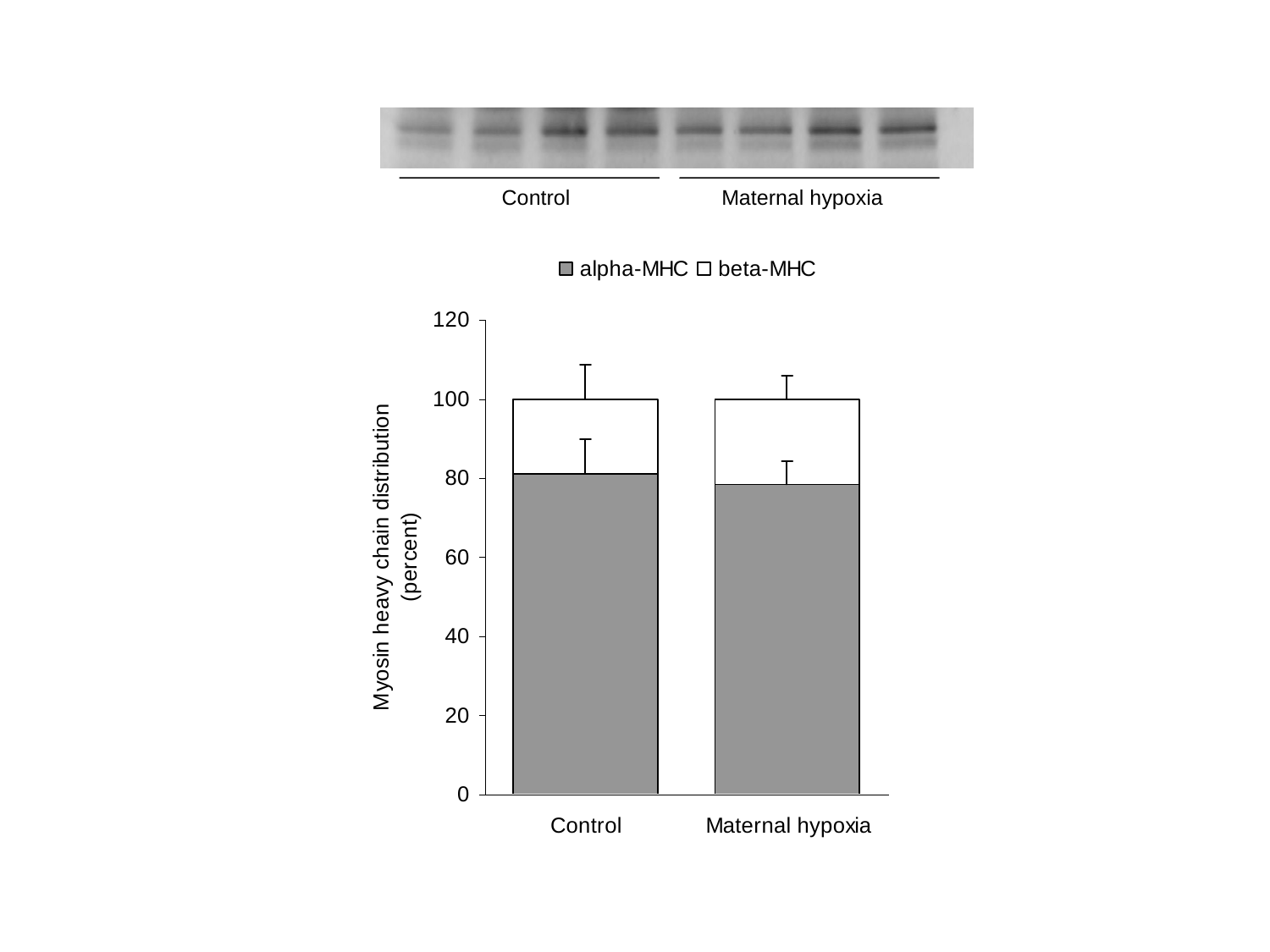

Control
Maternal hypoxia

Supplement: S5 Fig — Homogenates were isolated from left ventricle in a high PO4 = containing buffer and supernatants recovered. Samples were diluted using a non-denaturing, non-reducing sample buffer and loaded onto a polyacrylamide (6% w/v) gel containing glycerol (45% w/v) with 20mM pyrophosphate. Each sample corresponded to 0.5mg ventricle protein. Gels were run for 24hr at 4°C before staining with Coomassie Brilliant Blue Stain. For further details see Garcia et al. 2007 Eur. J. Physiol. 454 p.937–943. Densitometry was determined using computer software (ImageJ, NIH). Data represents Mean ± SD (n = 6). (PPTX) [file pone.0127424.s005.pptx]

## Slide 1
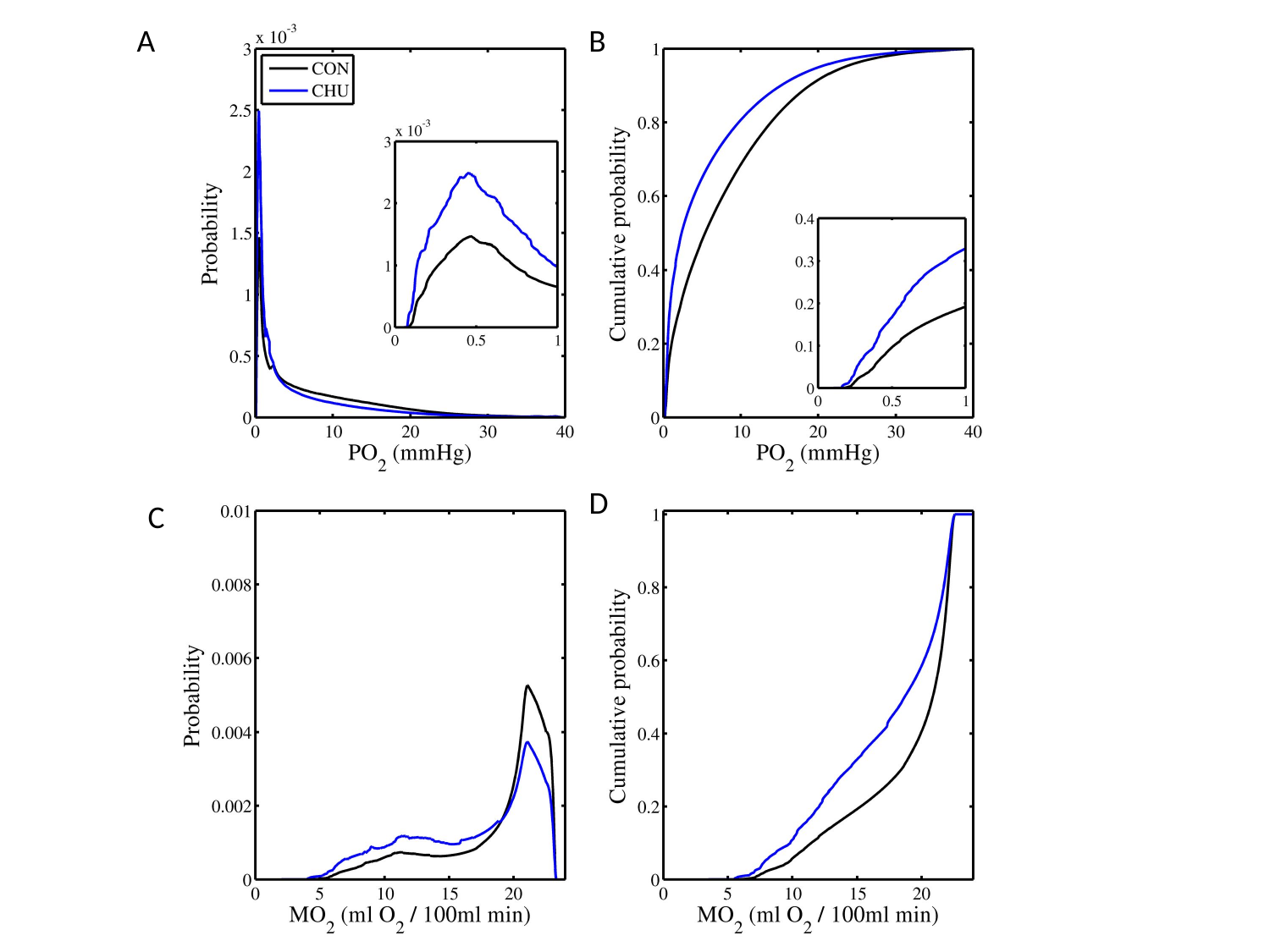

B
A
D
C

Supplement: S6 Fig — Probability (A) and cumulative probability (B) for tissue PO2 was calculated with reference to histological images to calculate domain area and citrate synthase activity to estimate the relative changes in tissue oxygen tension following CHU. Metabolic rate for individual cells (C) and cumulative probability (D) for metabolic rates were calculated from domain area and maximum predicted oxygen extraction measurements were used. For further details see methods. (PPTX) [file pone.0127424.s006.pptx]
