# Supplementary material for: Maternal Hypoxia Decreases Capillary Supply and Increases Metabolic Inefficiency Leading to Divergence in Myocardial Oxygen Supply and Demand
Source: PLoS One. 2015 Jun 1;10(6):e0127424. doi: 10.1371/journal.pone.0127424 (PMC4452690; doi:10.1371/journal.pone.0127424)
Supplement: S7 Fig — For further details see S1 Fig. Data represents mean ± SD (n = 6). Statistical significance represented: effects of hypoxia * P<0.05, ***P<0.001 (Paired students‘t’ test). Arterial blood gases were measured in pregnant rats to determine the effects of hypoxia (FIO2 = 0.12) on blood gases during pregnancy and, by extension, on the foetal environment. We note no changes to either pH or bicarbonate concentration in arterial plasma, with the greatest effect demonstrated as arterial hypoxaemia. The data indicate that we are justified in exploiting FIO2 as a mechanism to fix oxygen concentration without subsequent changes to plasma PaCO2, pH or bicarbonate concentrations. We demonstrate that anaesthesia has undoubtedly blunted arterial oxygen tension and led to relative hypercapnia compared with other evidence of hypocapnia as a consequence of pregnancy in humans [Moore et al. 1987 J. Appl. Physiol. 62 p.158–163, Lueder et al. 1995 Metabolism 44 p.532–537] and rats [Lueder et al. 1995 Metabolism 44 p.532–537]. (DOCX) [file pone.0127424.s007.docx]

| Measurement | Ambient Air (FIO_2_=0.2) | Hypoxia (FIO_2_=0.12) |
| --- | --- | --- |
| P_a_O_2_ (mmHg) | 90.5 ± 4.2 | 58.4 ± 2.0*** |
| P_a_CO_2_ (mmHg) | 41.0 ± 1.8 | 38.8 ± 2.9* |
| pH (units) | 7.39 ± 0.012 | 7.38 ± 0.034 |
| Bicarbonate (mM) | 24.9 ± 0.85 | 24.4 ± 0.60 |
